# A Hybrid Nonlinear Greater Cane Rat Algorithm with Sine–Cosine Algorithm for Global Optimization and Constrained Engineering Applications

**DOI:** 10.3390/biomimetics10090629

**Published:** 2025-09-17

**Authors:** Jinzhong Zhang, Anqi Jin, Tan Zhang

**Affiliations:** 1 School of Electrical and Photoelectronic Engineering, West Anhui University, Lu’an 237012, China; zhangjinzhongz@126.com (J.Z.); 42000028@wxc.edu.cn (T.Z.); 2 School of Electronics and Information Engineering, West Anhui University, Lu’an 237012, China

**Keywords:** greater cane rat algorithm, sine–cosine algorithm, nonlinear strategy, benchmark functions, engineering designs, exploration and exploitation

## Abstract

The greater cane rat algorithm (GCRA) is a swarm intelligence algorithm inspired by the discerning and intelligent foraging behavior of the greater cane rats, which facilitates mating during the rainy season and non-mating during the dry season. However, the basic GCRA exhibits serious drawbacks of high parameter sensitivity, insufficient solution accuracy, high computational complexity, susceptibility to local optima and overfitting, poor dynamic adaptability, and a severe curse of dimensionality. In this paper, a hybrid nonlinear greater cane rat algorithm with sine–cosine algorithm named (SCGCRA) is proposed for resolving the benchmark functions and constrained engineering designs; the objective is to balance exploration and exploitation to identify the globally optimal precise solution. The SCGCRA utilizes the periodic oscillatory fluctuation characteristics of the sine–cosine algorithm and the dynamic regulation and decision-making of nonlinear control strategy to improve search efficiency and flexibility, enhance convergence speed and solution accuracy, increase population diversity and quality, avoid premature convergence and search stagnation, remedy the disequilibrium between exploration and exploitation, achieve synergistic complementarity and reduce sensitivity, and realize repeated expansion and contraction. Twenty-three benchmark functions and six real-world engineering designs are utilized to verify the reliability and practicality of the SCGCRA. The experimental results demonstrate that the SCGCRA exhibits certain superiority and adaptability in achieving a faster convergence speed, higher solution accuracy, and stronger stability and robustness.

## 1. Introduction

The global optimization involves transforming complex problems with high-dimensionality, nonlinearity, and multiple constraints into explicit mathematical models with objective functions and constraints, within a defined variable domain or under specific constraints, which is utilized to regulate the quantitative indicators; attain the global extremum solution; acquire objective and repeatable results; eschew the subjectivity of empirical decision-making; and materialize optimal system performance, lowest cost, and highest efficiency. The traditional optimization methods, such as gradient descent, Newton’s method, Lagrange multiplier method, and simplex method, exhibit notable drawbacks: strong dependency on function properties, weak handling skills of high dimensions, sensitive constraint handling conditions, poor anti-interference and robustness, and narrow practical scenarios. However, metaheuristic algorithms (MHAs) exhibit remarkable advantages, including strong universality and usability, robustness and adaptability, excellent parallelism and distributed computing, extensive practicality and scalability, preferable detection efficiency and solution accuracy, and low computational complexity. MAs can be broadly categorized into four main types according to the source of inspiration to address the large-scale, nonlinear, multimodal optimization problems and identify the global extremum solutions.

(1) Swarm intelligence algorithms (SIAs)

SIAs, inspired by the collective behavior of organisms in nature, are distributed computing methods that imitate the local rules of numerous simple individuals to achieve interaction and collaboration, facilitating information sharing and adaptive adjustment, and thereby emerging with collective intelligent behavior, thereby efficiently acquiring the global extremum solution. SIAs exhibit local perception and global emergence, a balance of positive and negative feedback, a combination of randomness and determinism, and division of labor and collaboration mechanisms. SIAs exhibit specific competitive characteristics of substantial decentralization, self-organization, robustness, scalability, simplicity and ease implementation, excellent local interaction and global emergence, preferable distribution and parallelism, and reliable population diversity and global convergence, without gradient information, such as Chinese pangolin optimization (CPO) [1], black-winged kite algorithm (BKA) [2], elk herd optimization (EHO) [3], puma optimization (PO) [4], horned lizard optimization algorithm (HLOA) [5], and greater cane rat algorithm (GCRA) [6].

(2) Evolutionary algorithms (EAs)

EAs inspired by biological evolution theory are stochastic computational methods that imitate natural selection, genetic variation, and population reproduction. They utilize population iteration, fitness-driven, and random search mechanisms to approach global extremum solutions gradually. EAs are independent of the mathematical properties of continuity and differentiability of the problems, which are suitable for addressing nonlinear, non-convex, multimodal, high-dimensional, and multi-constraint problems. EAs exhibit specific competitive characteristics of intense exploration and exploitation, flexible multi-objective optimization and parallel computing, eschewing dimensional disaster and gradient information, strong flexibility, parallelism, scalability and adaptability, such as wave search algorithm (WSA) [7], snow avalanches algorithm (SAA) [8], liver cancer algorithm (LCA) [9], coronavirus mask protection algorithm (CMPA) [10], gooseneck barnacle optimization (GBO) [11], and orchard algorithm (OA) [12].

(3) Physics/Chemistry/Mathematics-inspired algorithms

Physics/Chemistry/Mathematics-inspired algorithms based on natural phenomena or mathematical principles are randomized computational methods, which imitate the energy conservation, gravitational interaction, thermodynamics process of physical systems, molecular interactions, energy conversion, chemical reaction equilibrium of the chemistry systems, mathematical transformations, probability distributions, and differential evolution of the mathematics systems to maintain population diversity and multi-objective dynamic equilibrium; promote information compensation coordination and collaborative optimization of dual populations; explore high-quality search regions; and exploit global extremum solutions. These algorithms exhibit specific competitive characteristics of strong multi-objective adaptability; parallelism and scalability; low parameter sensitivity; rigorous logicality and convergence; optimal complementary advantages; preferable universality and energy orientation; superior solution quality and efficiency, such as artemisinin optimization (AO) [13]; Newton–Raphson-based optimization (NRBO) [14]; exponential distribution optimizer (EDO) [15]; Young’s double-slit experiment (YDSE) [16]; Lévy arithmetic algorithm (LAA) [17]; and triangulation topology aggregation optimizer (TTAO) [18].

(4) Human-inspired algorithms (HBAs)

HBAs are inspired by social activities, human decision-making, cognitive patterns, environmental adaptation, and collaborative logic, which abstract the empirical rules, group interactions, adaptive strategies, or search logic accumulated by humans in addressing practical problems into computable mathematical models, and construct efficient search mechanisms. HBAs utilize individual experience accumulation, group information sharing, goal-oriented trial and error, or adaptive adjustment to efficiently explore the high-quality detection scope, extract global extremum solutions, achieve multi-agent collaboration, and avoid blind search and premature convergence. HBAs exhibit specific competitive characteristics of intense collaboration, adaptability, robustness, parallelism, interpretability and self-organization, straightforward interpretation and implementation; low dependency on mathematical properties; low computational complexity; intuitive and easily tunable parameters; strong multi-objective optimization and dynamic adaptability; and equilibrium between global exploration and local exploitation, such as educational competition optimization (ECO) [19], information acquisition optimizer (IAO) [20], human evolutionary optimization algorithm (HEOA) [21], memory backtracking strategy (MBS) [22], guided learning strategy (GLS) [23], and thinking innovation strategy (TIS) [24].

The greater cane rat algorithm (GCRA) is motivated by the dispersed foraging behavior during the mating season and the concentrated foraging behavior during the non-mating season of the greater cane rats, which facilitates an efficient switch between global exploration and local exploitation, expands the search range, and updates the population’s position to obtain potential optimal solutions [6]. The basic GCRA exhibits serious drawbacks of high parameter sensitivity, insufficient solution accuracy, high computational complexity, susceptibility to local optima and overfitting, poor dynamic adaptability, and severe curse of dimensionality. The no-free-lunch (NFL) theorem explicitly states that there is no universal algorithm or absolutely superior algorithm that applies to all complex problems, which not only reveals the conditional dependence of algorithms and the adaptability of difficulties but also prompts us to construct a hybrid nonlinear greater cane rat algorithm with sine–cosine algorithm called (SCGCRA) to resolve the benchmark functions and constrained engineering designs. The core purpose is to integrate the powerful global exploration ability of GCRA and the excellent local exploitation ability of SCA, and adaptively balance the two algorithms through a nonlinear control strategy, which can comprehensively improve the optimization performance of SCGCRA in handling complex function optimization and engineering practical problems, achieve dynamic balance between exploration and exploitation, enhance the adaptability and robustness of algorithms, provide efficient, reliable, and practical solutions for complex optimization problems. For function optimization, the SCGCRA employs mechanism complementarity, dynamic tuning, and information sharing to overcome local optima, accelerate convergence, and enhance solution accuracy. For engineering examples, the SCGCRA has certain superiority and practicality in handling complex constraints, adapting to dynamic environments, and solving high-dimensional problems. The SCGCRA not only employs global coarse exploration to enable greater cane rats to move and forage among scattered shelters within the territory, leave trail marks leading to food sources, and explore potential solutions, but also utilizes local refined exploitation to allow isolated males to concentrate on meticulous foraging in food-rich areas and enhance solution accuracy.

The main contributions of the SCGCRA are summarized as follows: (1) The hybrid nonlinear greater cane rat algorithm with sine–cosine algorithm (SCGCRA) is proposed to resolve the global optimization and constrained engineering applications. (2) The periodic oscillatory fluctuation characteristics of the sine–cosine algorithm and the dynamic regulation and decision-making of nonlinear control strategy improve search efficiency and flexibility, enhance convergence speed and solution accuracy, increase population diversity and quality, avoid premature convergence and search stagnation, remedy the disequilibrium between exploration and exploitation, achieve synergistic complementarity and reduce sensitivity, and realize repeated expansion and contraction. (3) The SCGCRA is compared with numerous advanced algorithms that contain recently published, highly cited, and highly performing algorithms, such as CPO, BKA, EHO, PO, WSA, HLOA, ECO, IAO, AO, HEOA, NRBO, and GCRA. (4) The SCGCRA is tested against twenty-three benchmark functions and six real-world engineering designs by performing simulation experiments and analyzing the results. (5) The evaluation metrics and overall performance of the SCGCRA outperform those of other algorithms. The SCGCRA exhibits substantial superiority and adaptability in achieving a dynamic balance between exploration and exploitation, leveraging a diversity mechanism to create a synergistic effect of complementary strengths, comprehensively enhancing convergence speed, solution accuracy, stability, and robustness.

The following sections constitute the article: Section 2 emphasizes the greater cane rat algorithm (GCRA). Section 3 elucidates the nonlinear greater cane rat algorithm with the sine–cosine algorithm (SCGCRA). Section 4 elaborates on the simulation test and result analysis for tackling benchmark functions. Section 5 portrays the SCGCRA for tackling engineering designs. Section 6 encapsulates the conclusions and future research.

## 2. Greater Cane Rat Algorithm (GCRA)

**Figure 1:**
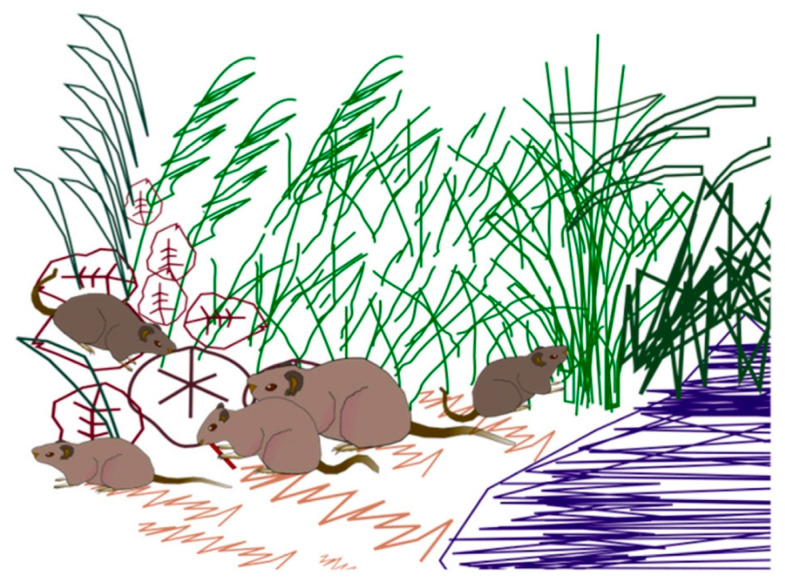
The natural foraging habitat of GCRs.

The GCRA is based on the intelligent foraging behavior and social collaboration mechanism of the greater cane rats (GCRs), which simulates their territory exploration, path tracking, and reproduction strategies to construct an efficient framework for global exploration and local exploitation in complex optimization problems. GCRs are highly nocturnal animals that primarily inhabit areas such as swamps, riverbanks, and cultivated land, with sugarcane and grass as their main diet. The GCRA simulates the dispersed foraging behavior of GCRs during the non-mating or dry season and the aggregated breeding behavior of GCRs during the mating or rainy season in balancing the ability to explore globally (search for unknown areas) and exploit locally (optimize known solutions). Figure 1 portrays the natural foraging habitat of GCRs. GCRs live near the water source in the bottom shaded areas. The whiteness of the regions and paths depicts the trails taken by the vine-like grasses, which are features of previously known food sources.

### 2.1. Population Initialization

The matrix of a randomly initialized population is calculated as follows:

(1) $$X=\left[\begin{array}{ccccc} x_{1,1} & x_{1,2} & \ldots & x_{1,d-1} & x_{1,d}\\ x_{2,1} & x_{2,2} & \ldots & x_{2,d-1} & x_{2,d}\\ \vdots & \vdots & x_{i,j} & \vdots & \vdots \\ x_{n,1} & x_{n,2} & \ldots & x_{n,d-1} & x_{n,d} \end{array}\right]$$

where X denotes the GCRs population, $x_{i,j}$ denotes ith location in jth dimension, n denotes the population scale, and d denotes the problem dimension. $x_{i,j}$ is calculated as follows:

(2) $$x_{i,j}=rand\;\times \;(UB_{j}-LB_{j})+LB_{j}$$

where rand∈[0,1] and UB and LB denotes the upper and lower boundaries.

The dominant male GCR $x_{k,j}$ is identified as the fittest individual, which can guide the group towards known food sources or shelters, realign the locations, and avoid blind search. ρ=0.5 serves as a variable that determines whether it is the rainy season or not, which is used to dynamically switch between exploration and exploitation.

(3) $$x_{i,j}^{new}=0.7\;\times \;\frac{\left(x_{i,j}+x_{k,j}\right)}{2}$$

where $x_{i,j}^{new}$ denotes the latest location and $x_{i,j}$ denotes the current location.

### 2.2. Exploration

**Figure 2:**
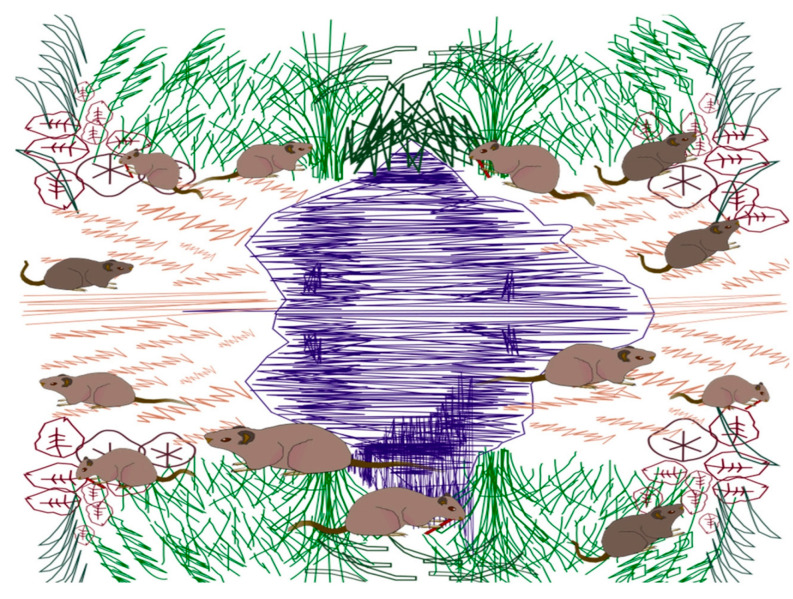
Exploration action of GCRs while looking for sources.

GCRs construct hideout or shallow burrow shelters scattered around the territory in swamps, riverbanks, and cultivated land, which achieves dispersed migration for foraging and leaves trail marks through territorial sheltering and route tracking. Figure 2 portrays the exploration action of GCRs while looking for sources. The most suitable location of the dominant male GCR is considered the food source route, while the remaining GCRs follow and adjust their locations to explore different areas, expand the solution regions, avoid search stagnation, and obtain multiple potential solutions. The location is calculated as follows:

(4) $$x_{i,j}^{new}=x_{i,j}+C\;\times \;(x_{k,j}-r\;\times \;x_{i,j})$$

(5) $$x_{i}=\left\{\begin{array}{c} x_{i,j}+C\;\times \;(x_{i,j}-\alpha \;\times \;x_{k,j}),\;\;\;\;\;\;\;F_{i}^{new}<F_{i}\\ x_{i,j}+C\;\times \;(x_{m,j}-\beta \;\times \;x_{k,j}),\;\;\;\;\;\;\;\;\mathrm{otherwise} \end{array}\right.$$

where $x_{i}$ denotes the latest location of ith GCR, $x_{i,j}^{new}$ denotes the location of jth dimension, $x_{i,j}$ denotes the GCR’s current location, $x_{k,j}$ denotes the dominant male GCR’s location, $F_{xk}$ denotes the fitness of the $x_{k,j}$, $F_{xi}$ denotes the current fitness, C∈[0,1] denotes the dispersed food sources and shelters, r imitates the impact of a diverse food source and intensify exploitation, α imitates a decreasing food source and forces GCRs to explore the latest food sources and shelters, and β promptes GCRs to relocate to other abundant food sources available within the breeding areas. r, α, and β are calculated as follows:

(6) $$r=F_{xk}-t\;\times \;(\frac{F_{xk}}{T})$$

(7) α=2 × r × rand−r

(8) β=2 × r × μ−r

where t denotes the current iteration and T denotes the maximum iteration.

### 2.3. Exploitation

**Figure 3:**
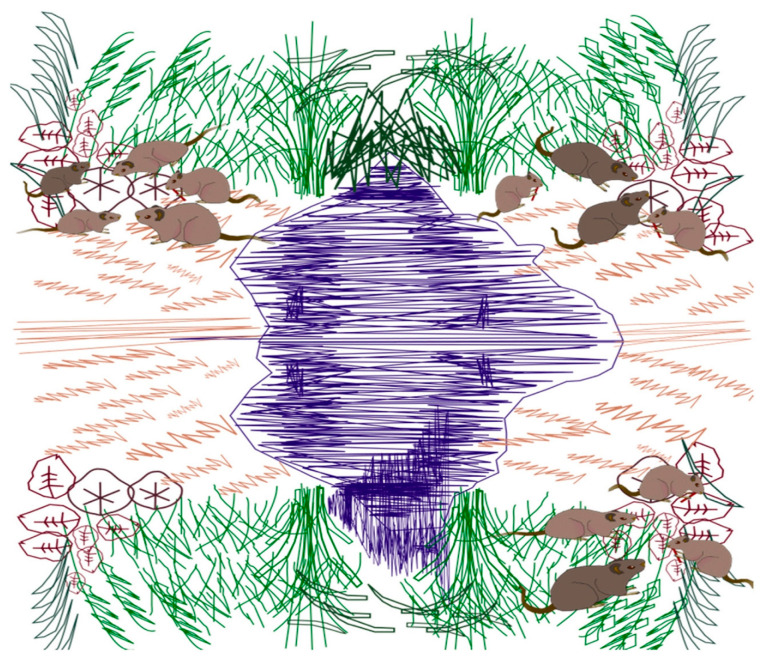
Exploitation action of GCRs during mating season.

Rampant breeding male GCRs leave the groups during the rainy season to forage deeply within areas with abundant food sources. Figure 3 portrays the exploitation action of GCRs during mating season. The GCRA utilizes the random selection of female GCRs to imitate the focused exploitation of high-quality areas through reproductive behavior, strength local meticulous search, and enhance solution quality. The location is calculated as follows:

(9) $$x_{i,j}^{new}=x_{i,j}+C\;\times \;(x_{k,j}-\mu \;\times \;x_{m,j})$$

where $x_{m,j}$ denotes the female GCR’s location and μ∈[1,4] imitates the number of offspring produced by each female GCR.

Algorithm 1 portrays the pseudocode of the GCRA.

**Algorithm 1** GCRA**Step 1.** Initialize the GCRs population $X_{i}(i=1,2,\ldots ,n)$
**Step 2.** Estimate the fitness of GCRs, renovate the global best solution (Gbest)
            Sift the fittest GCR as the dominant male $x_{k}$
            Renovate the remaining GCRs stem from $x_{k}$ via Equation (3)
**Step 3. while** t<T do
                  **for** all GCRs                            Renovate ρ, r, α, β, C, μ                            **if** rand<ρ                              **Exploration**                              Renovate GCRs positions via Equation (4)
                          **else**
                              **Exploitation**
                              Renovate GCRs positions via Equation (9)
                           **end if**
                    **end for**
                    Affirm whether any solution has overflowed the search interval and revise it
                    Estimate the fitness of GCRs stem from a renewed location
                     Renovate GCRs positions via Equation (5)
                     Renovate Gbest and sift a renewed dominant male $x_{k}$
                    t=t+1
            **end while**
            **Return** Gbest

## 3. Nonlinear Greater Cane Rat Algorithm with Sine–Cosine Algorithm (SCGCRA)

The SCGCRA integrates the intelligent discerning and foraging behavior of GCRA, the mathematical periodic oscillatory fluctuation characteristic of the sine–cosine algorithm, and the adaptive adjustment mechanism in remedying the disequilibrium between global exploration and local exploitation, enhancing convergence efficiency and solution accuracy, highlighting robustness and applicability, avoiding premature convergence and dimensional disaster, preventing redundant search, and achieving superior solution quality.

### 3.1. Nonlinear GCRA

The downward slope of the reduction in the parameter value has been altered according to the algorithm structure. The nonlinear control strategy exhibits a strong anti-disturbance ability and nonlinear processing characteristic, ensuring solution accuracy and stability, enhancing overall search efficiency and localized fine-tuning, strengthening adaptability and operability, and facilitating dynamic regulation and solution quality [25]. The locations are calculated as follows:

(10) $$W=2\cdot e^{-(\frac{8t}{T}{)}^{2}}$$

(11) $$x_{i,j}^{new}=W\;\times \;x_{i,j}+C\;\times \;(x_{k,j}-r\;\times \;x_{i,j})$$

(12) $$x_{i}=\left\{\begin{array}{c} W\;\times \;x_{i,j}+C\;\times \;(x_{i,j}-\alpha \;\times \;x_{k,j}),\;\;\;\;\;\;\;F_{i}^{new}<F_{i}\\ W\;\times \;x_{i,j}+C\;\times \;(x_{m,j}-\beta \;\times \;x_{k,j}),\;\;\;\;\;\;\;\;\mathrm{otherwise} \end{array}\right.$$

(13) $$x_{i,j}^{new}=W\;\times \;x_{i,j}+C\;\times \;(x_{k,j}-\mu \;\times \;x_{m,j})$$

where t denotes the current iteration and T denotes the maximum iteration.

### 3.2. Sine–Cosine Algorithm (SCA)

The SCA is derived from the periodic fluctuation and range restriction of sine and cosine functions, which imitates the dynamic oscillatory behavior of trigonometric functions to guide the search agents in efficiently searching within a multi-dimensional solution space and approximating the global optimal solution [26]. The SCA offers remarkable advantages, including concise structure and parameters, easy equilibrium and implementation, structural flexibility and stability, efficient astringency and solution quality, strong robustness and versatility, adaptive switching between exploration and exploitation, low computational overhead, and abundant population multiplicity. The location is calculated as follows:

(14) $$X_{i}^{t+1}=\left\{\begin{array}{c} X_{i}^{t}+r_{1}\;\times \;\sin(r_{2})\;\times \;\left|r_{3}\;\times \;P_{i}^{t}-X_{i}^{t}\right|,r_{4}<0.5\\ X_{i}^{t}+r_{1}\;\times \;\cos(r_{2})\;\times \;\left|r_{3}\;\times \;P_{i}^{t}-X_{i}^{t}\right|,\;r_{4}\geq 0.5 \end{array}\right.$$

where $X_{i}^{t}$ denotes the current location, $X_{i}^{t+1}$ denotes the latest location, $P_{i}^{t}$ denotes the fittest location, $r_{2}\in [0,2\pi ]$, $r_{3}\in [-2,2]$, $r_{4}\in [0,1]$, and ∣∣ denotes the absolute value.

The $r_{1}$ is calculated as follows:

(15) $$r_{1}=a-t\cdot \frac{a}{T}$$

where a=2.

### 3.3. SCGCRA

The SCGCRA utilizes the group migration mechanism led by the dominant male GCRs and the randomly dispersed food source simulation mechanism to provide clear global search directions, avoid direction dispersion and redundant detection caused by SCA’s mathematical periodic oscillatory fluctuation characteristics, ensure complete coverage of the solution space, dynamically expand the search scope, enhance population diversity, and avoid premature convergence. The SCGCRA utilizes the local aggregation mechanism of the female GCRs and narrow amplitude oscillations of the SCA to exploit a small range of high-quality solution areas, efficiently locate the optimal fitness scope, achieve weak fine adjustment, remedy local roughness, reduce ineffective iterations, accelerate the convergence speed, and enhance solution accuracy. The nonlinear control strategy can dynamically adjust the dispersion to avoid search stagnation and improve robustness and usability. The SCGCRA utilizes global guidance, local oscillation, and dynamic control to achieve a fast convergence speed, high solution accuracy, and strong anti-interference and adaptability.

In exploration of the SCGCRA, the location is calculated as follows:

(16) $$x_{i,j}^{{new}^{’}}=\left\{\begin{array}{c} x_{i,j}^{new}+r_{1}\;\times \;\sin(r_{2})\;\times \;\left|r_{3}\;\times \;x_{k,j}-x_{i,j}^{new}\right|,r_{4}<0.5\\ x_{i,j}^{new}+r_{1}\;\times \;\cos(r_{2})\;\times \;\left|r_{3}\;\times \;x_{k,j}-x_{i,j}^{new}\right|,r_{4}\geq 0.5 \end{array}\right.$$

(17) $${x_{i}}^{’}=\left\{\begin{array}{c} x_{i}+r_{1}\;\times \;\sin(r_{2})\;\times \;\left|r_{3}\;\times \;x_{k,j}-x_{i}\right|,r_{4}<0.5,F_{i}^{new}<F_{i}\\ x_{i}+r_{1}\;\times \;\cos(r_{2})\;\times \;\left|r_{3}\;\times \;x_{k,j}-x_{i}\right|,r_{4}\geq 0.5,F_{i}^{new}<F_{i}\\ x_{i}+r_{1}\;\times \;\sin(r_{2})\;\times \;\left|r_{3}\;\times \;x_{k,j}-x_{i}\right|,r_{4}<0.5,F_{i}^{new}\geq F_{i}\\ x_{i}+r_{1}\;\times \;\cos(r_{2})\;\times \;\left|r_{3}\;\times \;x_{k,j}-x_{i}\right|,r_{4}\geq 0.5,F_{i}^{new}\geq F_{i} \end{array}\right.$$

In exploitation of the SCGCRA, the location is calculated as follows:

(18) $$x_{i,j}^{{new}^{\prime \prime }}=\left\{\begin{array}{c} x_{i,j}^{new}+r_{1}\;\times \;\sin(r_{2})\;\times \;\left|r_{3}\;\times \;x_{k,j}-x_{i,j}^{new}\right|,r_{4}<0.5\\ x_{i,j}^{new}+r_{1}\;\times \;\cos(r_{2})\;\times \;\left|r_{3}\;\times \;x_{k,j}-x_{i,j}^{new}\right|,r_{4}\geq 0.5 \end{array}\right.$$

where $x_{i,j}^{new}$ denotes the location of jth dimension, $x_{i,j}^{new\prime }$ and $x_{i,j}^{new\prime \prime }$ denotes the latest location of jth dimension, $x_{i}$ denotes the location of ith GCR, $x_{i}^{’}$ denotes the latest location of ith GCR, $x_{k,j}$ denotes the dominant male GCR’s location, $r_{2}\in [0,2\pi ]$, $r_{3}\in [-2,2]$, $r_{4}\in [0,1]$, and $r_{1}$ is linearly decreases from 2 to 0.

The SCGCRA combines three existing techniques: GCRA, SCA, and a nonlinear control strategy. The motivation of a specific combination is summarized as follows: (1) Core problem-driven: Limitations of a single algorithm and the necessity of a hybrid design. The inherent characteristic of GCRA is to simulate the foraging and escape behavior of GCRs in terms of food source attraction and breeding season grouping, and achieve global detection and extensive search of the solution space. The GCRA lacks strong randomness and dispersion due to a lack of dynamic adjustment mechanisms, relying on individual experience accumulation and mathematically driven, refined development strategies, which determine limited solution accuracy. The inherent characteristics of SCA are based on the mathematical periodic oscillation characteristics of sine and cosine functions, which can systematically perform fine local development near the current optimal solution. The amplitude coefficient is usually linearly attenuated, and the later oscillation amplitude is too small to break through the local extremum. The SCGCRA achieves a balance between the breadth of global exploration and the accuracy of local exploitation by combining the biological behavior of GCRA with the mathematical periodicity of SCA, which has strong robustness and adaptability to high-dimensional, multimodal, and dynamic constraint problems. (2) Complementarity: A collaborative mechanism between biological behavior and mathematical models. The GCRA employs an adaptive grouping strategy and a constraint attraction mechanism to balance global exploration and local exploitation dynamically, guiding the population to move towards the feasible domain naturally. The SCA utilizes periodic oscillation coverage and amplitude dynamic attenuation to frequently switch directions, thereby enhancing the ability to escape local optima and requiring nonlinear control optimization. The SCGCRA possesses strong global exploration and local exploitation capabilities, enabling hybrid collaboration, identifying potential high-quality areas, and enhancing solution accuracy. (3) Nonlinear control strategy: Dynamic regulation and performance enhancement. The nonlinear control strategy adjusts SCGCRA parameters in real-time through feedback mechanisms, achieving optimal matching between solution quality and parameter adjustment. It avoids premature convergence or excessive oscillation caused by fixed parameters, ensuring the stability and robustness of the search process.

**Figure 4:**
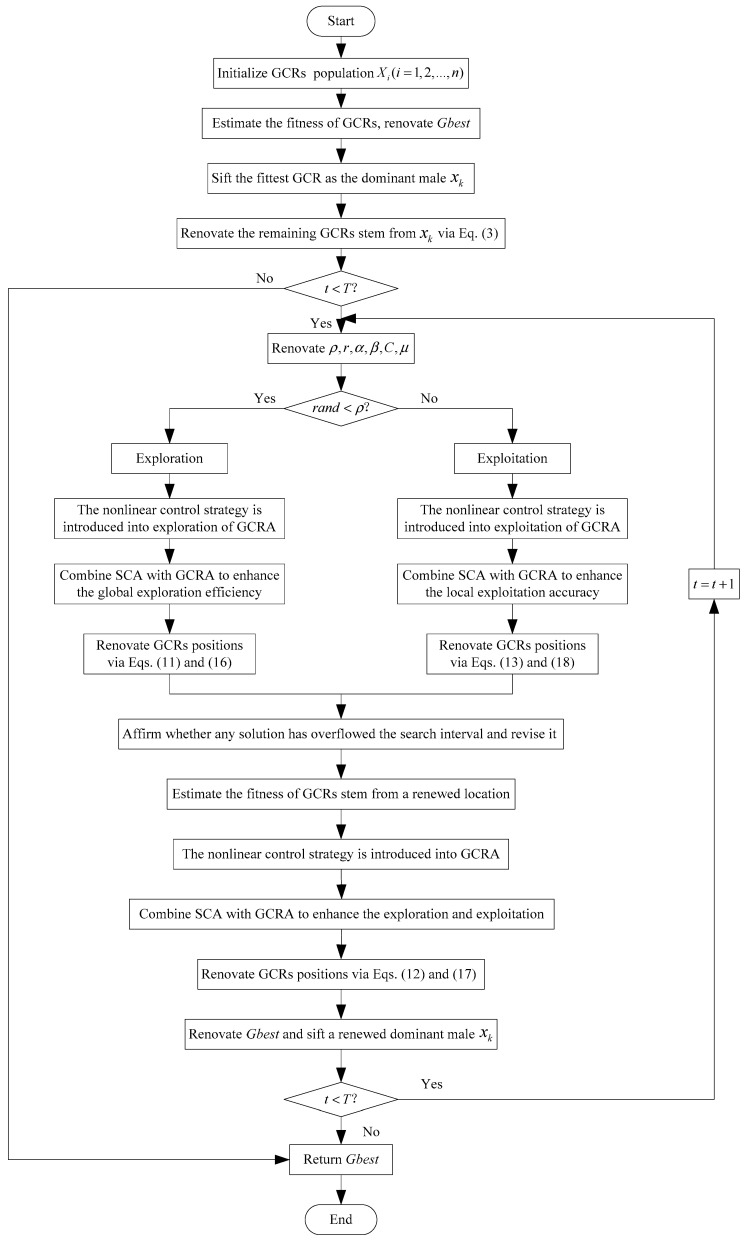
Flowchart of SCGCRA.

Algorithm 2 portrays the pseudocode of the SCGCRA. Figure 4 portrays the flowchart of SCGCRA.

**Algorithm 2** SCGCRA**Step 1.** Initialize the GCRs population $X_{i}(i=1,2,\ldots ,n)$
**Step 2.** Estimate the fitness of GCRs, renovate the global best solution (Gbest)
            Sift the fittest GCR as the dominant male $x_{k}$
            Renovate the remaining GCRs stem from $x_{k}$ via Equation (3)
**Step 3. while** t<T do
                  **for** all GCRs
                           Renovate ρ, r, α, β, C, μ
                           **if** rand<ρ
                               **Exploration**
                               The nonlinear control strategy is introduced into exploration of GCRA
                               Combine SCA with GCRA to enhance the global exploration efficiency
                               Renovate GCRs positions via Equations (11) and (16)
                           **else**
                               **Exploitation**
                               The nonlinear control strategy is introduced into exploitation of GCRA
                               Combine SCA with GCRA to enhance the local exploitation accuracy
                               Renovate GCRs positions via Equations (13) and (18)
                           **end if**
                     **end for**
                     Affirm whether any solution has overflowed the search interval and revise it
                     Estimate the fitness of GCRs stem from a renewed location
                     The nonlinear control strategy is introduced into GCRA
                     Combine SCA with GCRA to enhance the exploration and exploitation
                     Renovate GCRs positions via Equations (12) and (17)
                     Renovate Gbest and sift a renewed dominant male $x_{k}$
                     t=t+1
               **end while**
               **Return** Gbest

## 4. Simulation Test and Result Analysis for Tackling Benchmark Functions

### 4.1. Experimental Disposition

The experimental disposition stipulated a 64-bit Windows 11 OS, a 12th Gen Intel(R) Core(TM) i9-12900HX 2.30 GHz CPU, 4 TB storage, an independent 16 GB graphics card, and 16 GB RAM. All comparison approaches were implemented in MATLAB R2022b.

### 4.2. Benchmark Functions

The SCGCRA employed unimodal functions ($f_{1}-f_{7}$), multimodal functions ($f_{8}-f_{12}$), and fixed-dimension multimodal functions ($f_{13}-f_{23}$) to validate the reliability and practicality. Table 1 outlines the benchmark functions.

### 4.3. Parameter Settings

To validate the practicality and applicability, the SCGCRA is compared with the CPO, BKA, EHO, PO, WSA, HLOA, ECO, IAO, AO, HEOA, NRBO, and GCRA. The parameter selection and sensitivity analysis are summarized as follows: (1) Inheritance principle: The control parameters of SCGCRA are directly derived from the original parameters of GCRA and SCA, and strictly inherit the widely validated recommended or default values in the original papers. These parameters have been extensively studied and proven to possess broad applicability, reliable representativeness, and strong robustness. The necessity of repetitive experimental verification is relatively low. Modifying these highly standardized parameters would undermine the mathematical foundation and convergence guarantee of the SCGCRA. (2) Principles of Cybernetics: A nonlinear control strategy will smoothly transition and dynamically adjust the equivalent effects based on state variables, such as iteration times and population diversity, utilizing adaptive compensation mechanisms that are insensitive to the initial absolute values. A nonlinear control strategy can automatically compensate for performance losses caused by parameter deviations, ensure the robustness of parameter changes, theoretically guarantee the algorithm’s stability, and guide the search in a favorable direction. (3) Normalization principle: We dimensionless the few critical parameters that need to be set (such as values between 0 and 1, or correlated with the number of iterations), which dramatically reduces the coupling degree between selected parameters and the specific problem scale, making a set of parameters applicable to a class of problems. (4) Principle of structural superiority: The SCGCRA realizes the complementary advantages of the GCRA’s directional exploitation ability and SCA’s global exploration ability. The dynamic scheduling of the nonlinear control strategy makes the SCGCRA insensitive to subtle changes in parameters. The performance improvement primarily comes from structural innovation rather than fine-tuning of parameters. The algorithm structure itself ensures good performance within a reasonable range of parameters.

CPO: Invariable values Q=100, Dc=0.6, β=1.5.

BKA: Stochastic values rand∈[0,1], r∈[0,1], Cauchy mutation C∈(0,1), invariable values p=0.9, δ=1, μ=0.

EHO: Stochastic values α∈[0,1], β∈[0,2], γ∈[0,2].

PO: Invariable values $PF_{1}=0.5$, $PF_{2}=0.5$, $PF_{3}=0.3$, U=0.2, stochastic values L∈[0.7,0.9], α∈[1,2].

WSA: Invariable values $a_{0}=0.3$, c=1.6.

HLOA: Hue circle angle h∈[0,2π], binary value σ=0   or   1, invariable values ∂=2, $v_{0}=1$, α=π/2, $\varepsilon =1\;\times \;10^{-6}$, g=0.009807, stochastic values Light∈[0,0.4046661], Dark∈[0.5440510,1], walk∈[−1,1].

ECO: Stochastic values $R_{1}\in [0,1]$, $R_{2}\in [0,1]$, invariable values γ=1.5, H=0.5, E=1.

IAO: Stochastic values ϑ∈[0,1], rand∈[0,1], v∈[0,1], β∈[0,1], γ∈[0,1], δ∈[0,1], ε∈[0,1], ζ∈[0,1], κ∈[0,1], w∈[0,1].

AO: Stochastic values R∈[0,1], $r_{1}\in [0,1]$, d∈[0.1,0.6].

HEOA: Stochastic values rand∈(0,1), R∈[0,1], invariable values γ=1.5, A=0.6.

NRBO: Stochastic values rand∈(0,1), δ∈[−1,1], a∈(0,1), b∈(0,1), $r_{1}\in (0,1)$, $r_{2}\in (0,1)$, $\theta_{1}\in (-1,1)$, $\theta_{2}\in (-0.5,0.5)$, Δ∈(0,1), invariable value DF=0.6, binary value β=0   or   1.

GCRA: Stochastic values rand∈(0,1), C∈[0,1], μ∈[1,4], invariable value ρ=0.5.

SCGCRA: Stochastic values rand∈(0,1), C∈[0,1], μ∈[1,4], $r_{2}\in [0,2\pi ]$, $r_{3}\in [-2,2]$, $r_{4}\in [0,1]$, invariable values ρ=0.5, a=2.

### 4.4. Simulation Test and Result Analysis

To objectively and comprehensively evaluate the convergence characteristics and solution accuracy of each algorithm, the population scale, maximum iteration, and stand-alone run remained consistent, with values of 50, 1000, and 30, respectively. Table 2 outlines the contrastive results of benchmark functions.

The SCGCRA was manipulated to resolve the benchmark functions; the core objective was to break through the limitations of the original GCRA and the bottlenecks of local optimum, enhance the adaptability and reliability of strongly constrained functions, promote the detection efficiency and solution quality, strengthen stability and robustness, diminish the parameter sensitivity and result fluctuations, and identify the global optimum or the high-quality approximate solution. The optimal value (Best), worst value (Worst), mean value (Mean), and standard deviation (Std) systematically validate the core characteristics and reflect the reliability and applicability from different dimensions. The optimal value is the objective fitness value corresponding to the fittest solution in multiple stand-alone runs, which verifies the global detectability and convergence accuracy. When the optimal value approaches the theoretical global optimum, the algorithm has enhanced the potential to excavate high-quality solutions and strengthened the effectiveness to break through the local optimum. The rapid decrease and stabilization of the optimal value during the iteration process indicate that the algorithm has a fast convergence speed and high solution accuracy. The worst value is the objective fitness value corresponding to the worst solution in multiple stand-alone runs, which estimates the robustness and adaptability to extreme scenarios. The smaller the disparity between the worst value and the optimal value, the lower the sensitivity of the algorithm to initial population, parameter settings, complex solution domain, or randomness, and the stronger the robustness. The modified strategy significantly surpasses the original algorithm in terms of value. It effectively reduces the number of extreme poor solutions during the search process, thereby avoiding infeasible solutions and extreme local convergence. The mean value is the arithmetic mean of the objective fitness values of all solutions in multiple stand-alone runs, which reveals the overall average performance, search efficiency, and general applicability. It assesses the stability of the algorithm and the uniformity of the distribution of the disaggregation. The extent to which the mean value approaches the theoretical optimal solution evaluates the overall search efficiency of the algorithm, designates the correctness of the overall search direction, and avoids search stagnation due to local optima. The standard deviation is the degree of the objective fitness value relative to the mean value, which measures stability and distribution uniformity of the algorithm. The smaller standard deviation indicates that the algorithm exhibits less fluctuation in the convergence results, higher stability, and stronger repeatability. The standard deviation is extended to the degree of discrepancy in the objective space, which is used to evaluate the impact of the modified approach on solution diversity. For unimodal functions, the solution space comprises a unique global extremum point without local extremum points. The monotonicity can swiftly narrow the search scope, diminish ineffective exploration, and clarify the direction of gradient descent. The core requirement was to rapidly and precisely approximate the optimal solution, which validates the algorithm’s local mining accuracy, convergence efficiency, and adaptability of simple solution spaces. For $f_{1}$, $f_{2}$, $f_{3}$, and $f_{4}$, the optimal values, worst values, mean values, and standard deviations of the IAO, GCRA, and SCGCRA remained consistent and exhibited optimal extreme solutions. The quantitative metrics, detection efficiency, and solution accuracy of the SCGCRA were superior to those of the CPO, BKA, EHO, PO, WSA, HLOA, ECO, AO, HEOA, and NRBO, and the SCGCRA utilized the aggregation effect of dominant male GCRs and the decentralized search of the population to overlap the global exploration scope, locate potential optimal areas, strengthen local exploitation, and enhance solution accuracy. For $f_{5}$, $f_{6}$, and $f_{7}$, the SCGCRA not only achieved a small improvement in quantitative metrics, detection efficiency, and solution accuracy but also significantly outperformed other algorithms. The SCGCRA utilized the periodic fluctuations of sine and cosine functions to furnish multi-directional perturbation paths, actualize high-precision local convergence, avert search oscillation, narrow the search solution space, and ensure population diversity. For multimodal functions $f_{8}-f_{12}$, the solution space comprised multiple local extremum points, some of which were close in solution quality to the global extremum point. The core requirement was to avoid search stagnation, surmount local traps, and identify the global optimum, which validates the algorithm’s global search capability, the maintenance and activation of population diversity. For $f_{8}$ and $f_{10}$, the optimal values, worst values, mean values, and standard deviations of the CPO, BKA, PO, WSA, HLOA, ECO, IAO, NRBO, and GCRA, the SCGCRA remained consistent and exhibited optimal extreme solutions, which were superior to those of the EHO and AO. The SCGCRA integrated the nonlinear control strategy of GCRA and the periodic oscillatory fluctuation of SCA to guide dominant individuals, adjust dynamic step sizes, and enhance adaptability and complementarity. For $f_{9}$, $f_{11}$, and $f_{12}$, the quantitative metrics, detection efficiency, and solution accuracy of the SCGCRA were better than those of the CPO, BKA, EHO, PO, WSA, HLOA, ECO, IAO, AO, HEOA, NRBO, and GCRA. The SCGCRA prioritized strong reliability and practicality in scanning the solution scope, facilitating extensive global exploration, enhancing group collaboration and guidance, discovering precise optimal solutions, and avoiding premature convergence. For fixed-dimension multimodal $f_{13}-f_{23}$, the solution space retained multiple local extremum points of multimodal functions and a unique global extremum point of unimodal functions, adopted fixed-dimensionality to control complexity, and eliminated interference of dimensionality changes. The core requirement was to resist performance degradation caused by increased dimensionality, which validates the algorithm’s global stability, robustness, and consistency, the decoupling and search direction focusing on high-dimensional variables, adaptability to the curse of dimensionality, and equilibrium ability between detection and exploitation. For $f_{13}$, $f_{14}$, $f_{15}$, $f_{16}$, $f_{17}$, $f_{21}$, $f_{22}$, and $f_{23}$, the optimal values, worst values, and mean values of the SCGCRA remained consistent and exhibited optimal extreme solutions; the quantitative metrics, detection efficiency, and solution accuracy of the SCGCRA were superior to those of CPO, BKA, EHO, PO, WSA, HLOA, ECO, IAO, AO, HEOA, NRBO, and GCRA. The SCGCRA exhibited strong stability and robustness in effectively guiding and regulating the initial population distribution, reducing dependence on initial conditions, and steadily searching towards the optimal solution, and diminishing the algorithm’s variability. For $f_{18}$, $f_{19}$, and $f_{20}$, the SCGCRA exhibited strong superiority and operability in terms of the quantitative metrics, detection efficiency, and solution accuracy. The SCGCRA utilized the random fluctuation and periodicity of SCA, the group collaboration and guidance mechanism of GCRA, and dynamic adjustment and search mechanism of the nonlinear control strategy to explore the solution scope extensively and intensively identify potential high-quality regions; meticulously exploit the extremum solutions; effectively avoid blind large-scale search; strictly avert search stagnation and slow convergence; and preferably balance exploration and exploitation to approximate the optimal solution.

### 4.5. Convergence Analysis

**Figure 5:**
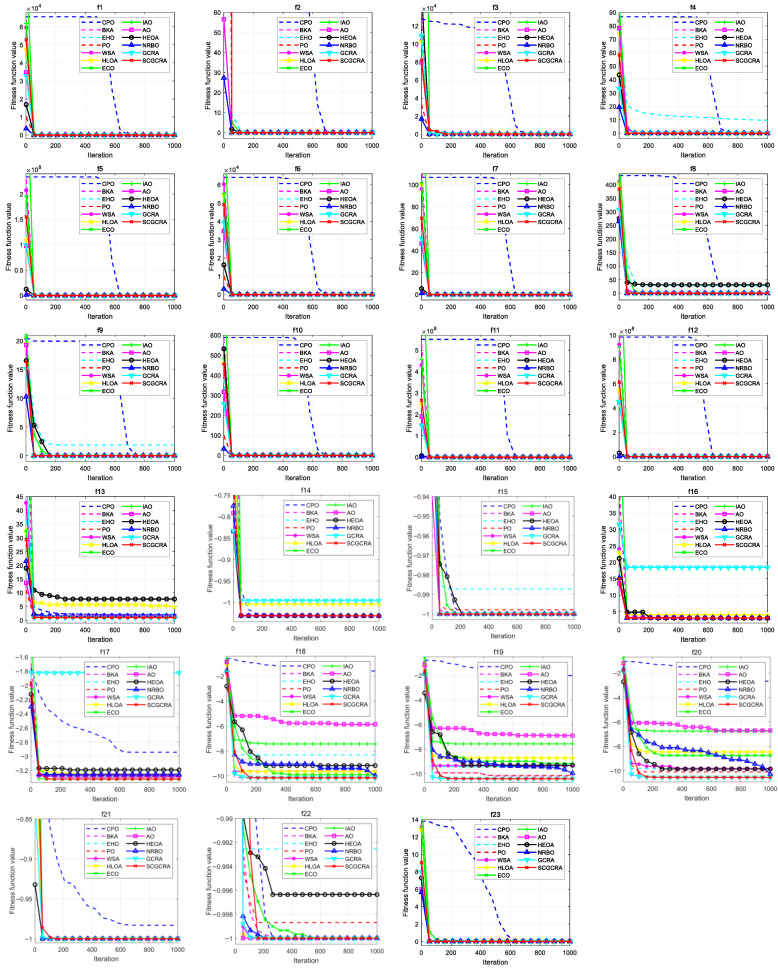
Convergence curves of the SCGCRA and comparative algorithms for addressing the benchmark functions.

Figure 5 portrays the convergence curves of the SCGCRA and comparative algorithms for addressing the benchmark functions. The convergence curves can be used to determine whether an algorithm can efficiently approximate the optimal solution and measure solution accuracy by observing steep descent, plateau periods, and oscillatory curves. This also allows for quantifying convergence efficiency by observing the number of iterations required to reach a specific stable value quickly. The optimal value and mean value jointly reveal the search efficiency and convergence accuracy of the algorithm. If the disparity between the optimal value and the average value is significant, it validates that the algorithm has prematurely fallen into a local optimum. If the discrepancy between the optimal value and the average value is slight and continues to decrease, it validates that the algorithm exhibits a strong equilibrium between global exploration and local exploitation. For unimodal functions, the numerous quantitative metrics, detection efficiency, and solution accuracy of the SCGCRA were superior to those of the CPO, BKA, EHO, PO, WSA, HLOA, ECO, IAO, AO, HEOA, NRBO, and GCRA. The SCGCRA utilized the territorial foraging behavior and dominant individual guidance mechanism of the GCRA to quickly locate potential optimal areas, avoid aimless search, cover the solution space, remedy the disequilibrium between exploration and exploitation, and enhance robustness and reliability. For multimodal functions, the SCGCRA exhibited remarkable advantages and superiority in terms of numerous quantitative metrics, detection efficiency, and solution accuracy compared to other comparative algorithms. The SCGCRA utilized the mathematical periodic oscillatory fluctuation of the SCA to enhance local exploitation, quantify solution accuracy, avoid dimensional disaster, achieve synergistic complementarity, and reduce sensitivity, thereby increasing population diversity and flexibility. For fixed-dimensional multimodal $f_{13}-f_{23}$, the multitudinous optimal values, mean values, detection efficiency, and solution accuracy of the SCGCRA outperformed the comparative algorithms. The SCGCRA employed a nonlinear control strategy to exhibit strong anti-disturbance ability and stability, thereby enhancing adaptability, operability, and practicality; facilitating repeated expansion and contraction; and improving dynamic regulation and solution quality. To summarize, the SCGCRA integrated the periodic oscillatory fluctuation characteristics of the SCA and the dynamic regulation and decision-making of a nonlinear control strategy to provide clear global search directions, avoid direction dispersion and redundant detection, ensure complete coverage of the solution space, enhance population diversity, and achieve good detection efficiency and solution accuracy.

### 4.6. Boxplot Analysis

**Figure 6:**
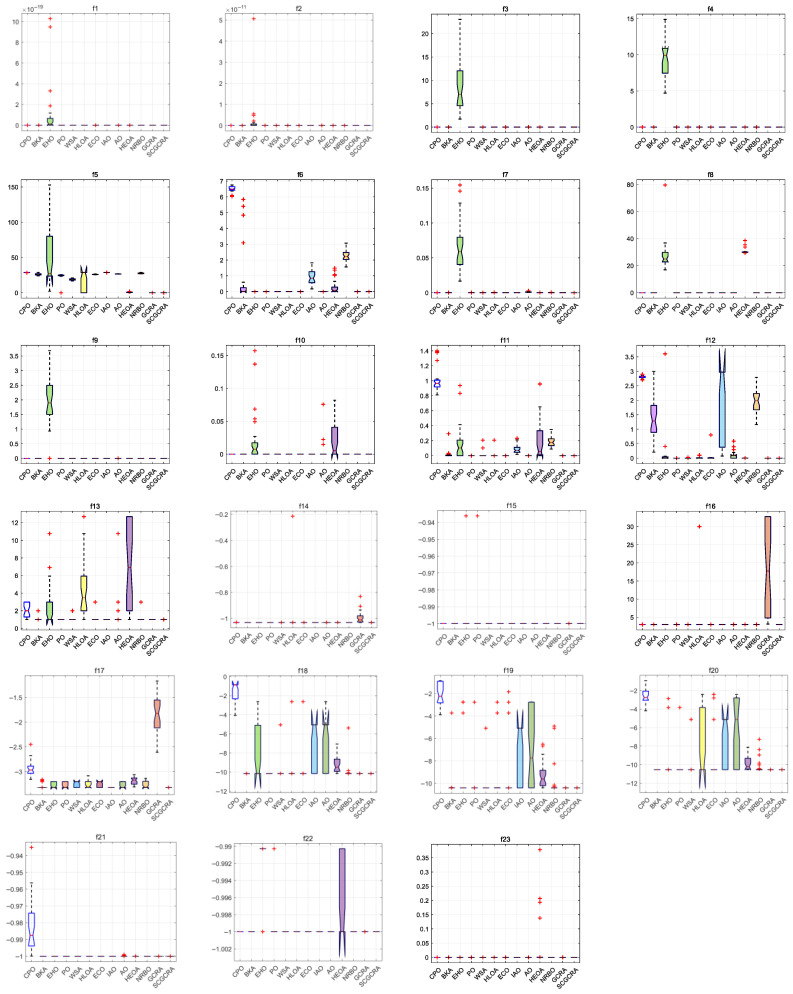
Boxplots of the SCGCRA and comparative algorithms for addressing the benchmark functions.

Figure 6 portrays boxplots of the SCGCRA and comparative algorithms for addressing the benchmark functions. The boxplots can quantify the stability and reliability of solutions, reflect the changes in solution diversity over time, and describe the dispersion of individual solutions within the population. The standard deviation intuitively demonstrates the sensitivity of the algorithm to initial population and parameter perturbations. The continuous decrease in standard deviation indicates that the algorithm maintains good convergence to force the population to converge towards the global extremum solution. If standard deviation mutates or oscillates, it may fall into a local optimum or require parameter adjustment. The standard deviation and worst value jointly reveal the stability and robustness of the algorithm. If the standard deviation is slight, the worst value infinitely approaches the optimal value, which validates that the algorithm is insensitive to randomness and the adaptability of initial conditions. The standard deviation has strong stability and expansibility in assessing the uniformity of the distribution of the solution set (such as the dispersity of solutions on the Pareto front in multi-objective optimization) and in verifying the profound impact of improvement strategies on solution diversity. For unimodal functions $f_{1}-f_{7}$, the multitudinous standard deviation and dispersion of the SCGCRA were superior to those of other comparative algorithms. The SCGCRA exhibited strong adaptability and operability in overcoming the drawbacks of high parameter sensitivity, insufficient solution accuracy, high computational complexity, susceptibility to local optima and overfitting, poor dynamic adaptability, and the severe curse of dimensionality. For multimodal functions $f_{8}-f_{12}$, compared with other comparative algorithms, the SCGCRA exhibited remarkable advantages and superiority in terms of the multitudinous standard deviation and dispersion. The SCGCRA utilized the intelligent foraging behavior and social collaboration mechanism of GCRs to simulate territory exploration, path tracking, and reproduction strategies. The SCGCRA employed a nonlinear control strategy to ensure solution accuracy and stability, facilitate dynamic regulation, and improve solution quality. For fixed-dimensional multimodal $f_{13}-f_{23}$, the multitudinous standard deviation and dispersion of the SCGCRA outperformed the comparative algorithms. The SCGCRA exhibited strong reliability and practicality, facilitating mating during the rainy season and non-mating during the dry season. This enabled the expansion of the search range, updated population positions, and enhanced population diversity, ultimately identifying the globally optimal precise solution. To summarize, the SCGCRA not only exhibited substantial superiority and adaptability to determine the superior standard deviation and dispersion and enhance the stability and robustness, but also exhibited strong practicality and reliability to balance the global coarse exploration and local refined exploitation, overlap the global exploration scope, locate potential optimal areas, strengthen local exploitation, and enhance solution accuracy.

### 4.7. Wilcoxon Rank-Sum Test

The Wilcoxon rank-sum test is a non-parametric statistical approach for paired data, which quantifies whether the overall discrepancy between the SCGCRA and other algorithms is statistically significant without relying on assumptions about data distribution [27]. p<0.05 designates the dramatic difference, p≥0.05 designates the non-dramatic difference, and N/A designates “not applicable”. Table 3 portrays the contrastive results of the *p*-value Wilcoxon rank-sum test on the benchmark functions. The SCGCRA exhibits strong stability and reliability in acquiring genuine and effective data rather than accidental data.

## 5. SCGCRA for Tackling Engineering Designs

To validate adaptability and practicality, the SCGCRA was utilized to tackle the constrained real-world engineering designs: three-bar truss design [28], piston lever design [29], gear train design [30], car side impact design [31], multiple-disk clutch brake design [32], and rolling element bearing design [33].

### 5.1. Three-Bar Truss Design

**Figure 7:**
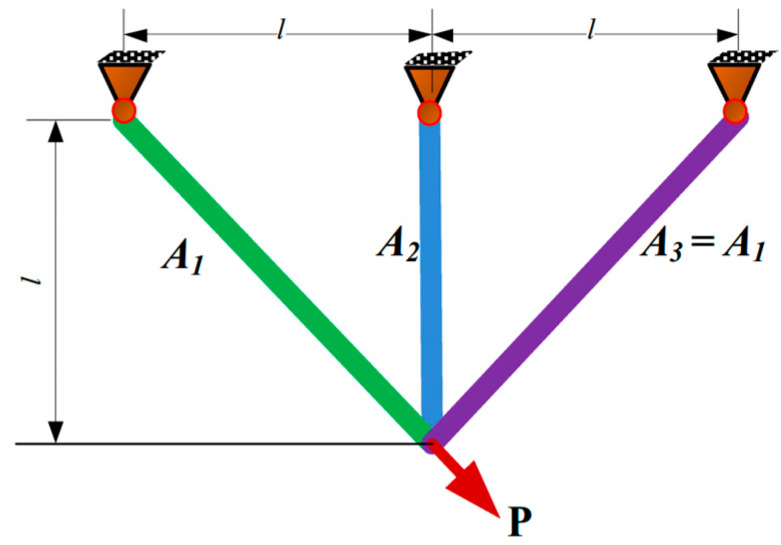
Sketch map of the three-bar truss design.

The dominant motivation was to weaken the cumulative weight, which incorporated two quantitative metrics: the intersecting surfaces $A_{1}$ and $A_{2}$. Figure 7 portrays the sketch map of the three-bar truss design.

Consider

(19) $$x=[x_{1}\;\;\;x_{2}]=[A_{1}\;\;\;A_{2}]$$

Minimize

(20) $$f(x)=(2\sqrt{2}x_{1}+x_{2})\;\times \;l$$

Subject to

(21) $$g_{1}(x)=\frac{\sqrt{2}x_{1}+x_{2}}{\sqrt{2}x_{1}^{2}+2x_{1}x_{2}}P-\sigma \leq 0$$

(22) $$g_{2}(x)=\frac{x_{2}}{\sqrt{2}x_{2}+2x_{1}x_{2}}P-\sigma \leq 0$$

(23) $$g_{3}(x)=\frac{1}{\sqrt{2}x_{2}+x_{1}}P-\sigma \leq 0$$

(24) $$l=100\;cm,\;\;\;\;\;\;P=2\;kN/cm^{2},\;\;\;\;\;\;\sigma =2\;kN/cm^{2}$$

Variable range

(25) $$0\leq x_{1},x_{2}\leq 1$$

Table 4 outlines the contrastive results of the three-bar truss design. The SCGCRA used periodic oscillations of sine and cosine functions to achieve large-scale and multi-directional coverage of the solution space without relying on neighborhood continuity. This can directly cross distant subregions in high-dimensional space, avoiding the traversal blind spots caused by a single GCRA dominated by local neighborhood search. The SCGCRA employed a large step size for strong fluctuation and a small step size for weak contraction to lock out the high-quality regions, approximate the computational extreme solution, weaken redundant searches, and accelerate convergence speed. The global extremum solution was materialized by the SCGCRA at quantitative metrics: 0.78645 and 0.41813, with the optimum weight of 263.8543.

### 5.2. Piston Lever Design

**Figure 8:**
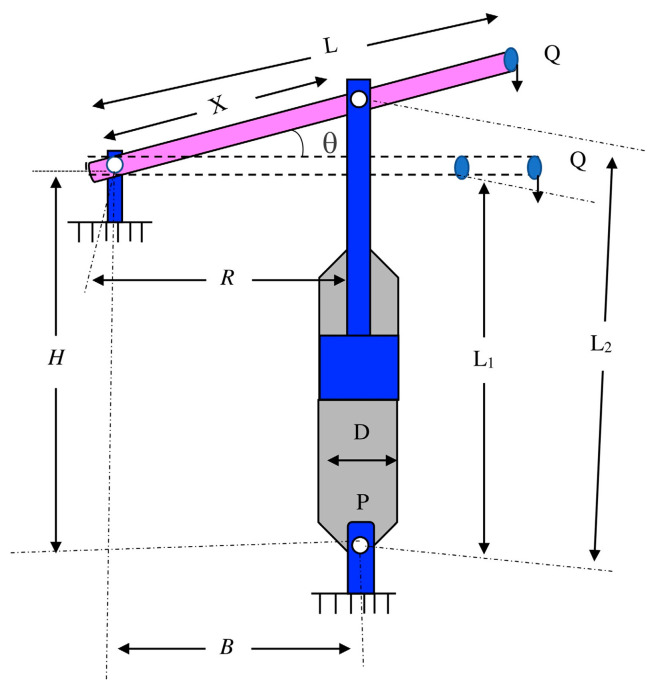
Sketch map of the piston lever design.

The dominant motivation was to weaken (attenuate) the cumulative weight, oil volume, and ascertain the components as the piston joystick is elevated from 0° to 45°, which incorporated four quantitative metrics: H, B, X, and D. Figure 8 portrays the sketch map of the piston lever design.

Consider

(26) $$x=[x_{1}\;\;\;x_{2}\;\;\;x_{3}\;\;\;x_{4}]=[H\;\;\;B\;\;\;D\;\;\;X]$$

Minimize

(27) $$f(x)=\frac{1}{4}\pi x_{3}^{2}(L_{2}-L_{1})$$

Subject to

(28) $$g_{1}(x)=QL\cos\theta -RF\leq 0$$

(29) $$g_{2}(x)=Q(L-x_{4})-M_{\max}\leq 0$$

(30) $$g_{3}(x)=\frac{6}{5}\;\times \;(L_{2}-L_{1})-L_{1}\leq 0$$

(31) $$g_{4}(x)=\frac{x_{3}}{2}-x_{2}\leq 0$$

(32) $$R=\frac{\left|-x_{4}(x_{4}\sin\theta +x_{1})+x_{1}(x_{2}-x_{4}\cos\theta )\right|}{\sqrt{{\left(x_{4}-x_{2}\right)}^{2}+x_{1}^{2}}}$$

(33) $$F=\frac{\pi Px_{3}^{2}}{4}$$

(34) $$L_{1}=\sqrt{{\left(x_{4}-x_{2}\right)}^{2}+x_{1}^{2}}$$

(35) $$L_{2}=\sqrt{{\left(x_{4}\sin\theta +x_{1}\right)}^{2}+{\left(x_{2}-x_{4}\cos\theta \right)}^{2}}$$

(36) $$\theta =45{}^{\circ},\;\;\;Q=10000\;lbs,\;\;\;L=240\;in,\;\;\;M_{\max}=1.8\;\times \;10^{6}\;lbs\;\;in,\;\;\;P=1500\;psi$$

Variable range

(37) $$0.05\leq x_{1},x_{2},x_{4}\leq 500,\;\;\;\;\;\;0.05\leq x_{3}\leq 120$$

Table 5 outlines the contrastive results of the piston lever design. The SCGCRA simulated the regional search and information sharing behavior of GCRs, which adopts individual position interaction and group optimal guidance to systematically cover the multimodal space of nonlinear problems, fully detect globally, accurately mine locally, and balance optimization efficiency and stability. The SCGCRA utilized the periodic oscillatory fluctuations of the SCA to overcome the exploration limitations of a population converging towards dominant individuals, ensure the population focuses on nuanced exploration in high-quality areas, and avoid the step size from rigidly oscillating near the extremal solution. The global extremum solution was materialized by the SCGCRA at quantitative metrics: 0.05, 0.125364154, 120, and 4.12410157, with the optimum weight of 7.794.

### 5.3. Gear Train Design

**Figure 9:**
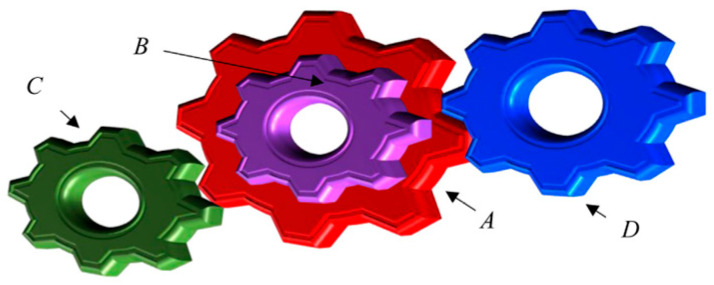
Sketch map of the gear train design.

The dominant motivation was to weaken (attenuate) the cumulative teeth size and the gear ratio’s optimum cost, which incorporates four quantitative metrics: the teeth scales of the gear train $n_{A}$, $n_{B}$, $n_{C}$ and $n_{D}$. Figure 9 portrays the sketch map of the gear train design.

Consider

(38) $$x=[x_{1}\;\;\;x_{2}\;\;\;x_{3}\;\;\;x_{4}]=[n_{A}\;\;\;n_{B}\;\;\;n_{C}\;\;\;n_{D}]$$

Minimize

(39) $$f(x)={\left(\frac{1}{6.931}-\frac{x_{3}x_{2}}{x_{1}x_{4}}\right)}^{2}$$

Variable range

(40) $$12\leq x_{i}\leq 60,\;\;\;i=1,2,\ldots ,4$$

Table 6 outlines the contrastive results of the gear train design. The SCGCRA utilized the sine direction adjustment and cosine step size control to achieve periodic small-scale fluctuations within the potential optimal region locked by GCRA, dynamically decreased the step size with iteration, and compensated for the shortcomings of slow convergence speed and low solution accuracy of a single GCRA in the later stage. The SCGCRA exhibited strong adaptability and practicality, facilitating the exploration of fluctuations and targeted exploitation, thereby covering the solution space, triggering population diversity, avoiding chaotic search, and enhancing resistance to the curse of dimensionality. The SCGCRA materialized the global extremum solution at quantitative metrics: 50, 22, 19, and 52, with the optimum cost of 3.25$\;\times \;10^{-18}$.

### 5.4. Car Side Impact Design

**Figure 10:**
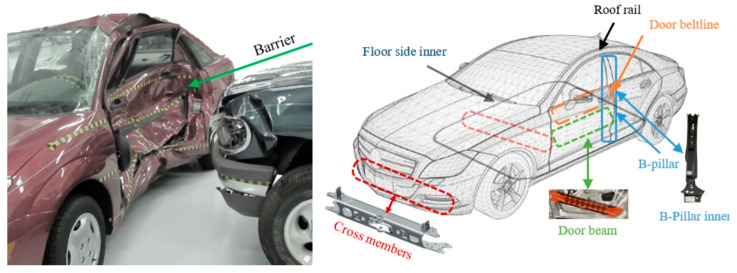
Sketch map of the car side impact design.

The dominant motivation was to weaken (attenuate) the cumulative weight, which incorporates 11 quantitative metrics: thicknssssess of B-pillar inner ($x_{1}$), B-pillar reinforcement ($x_{2}$), floor side inner ($x_{3}$), cross members ($x_{4}$), door beam ($x_{5}$), door beltline reinforcement ($x_{6}$), roof rail ($x_{7}$), materials of B-pillar inner ($x_{8}$), floor side inner ($x_{9}$), barrier height ($x_{10}$), and hitting position ($x_{11}$). Figure 10 portrays the sketch map of the car side impact design.

Consider

(41) $$x=[x_{1}\;\;\;x_{2}\;\;\;x_{3}\;\;\;x_{4}\;\;\;x_{5}\;\;\;x_{6}\;\;\;x_{7}\;\;\;x_{8}\;\;\;x_{9}\;\;\;x_{10}\;\;\;x_{11}]$$

Minimize

(42) $$f(x)=1.98+4.90x_{1}+6.67x_{2}+6.98x_{3}+4.01x_{4}+1.78x_{5}+2.73x_{7}$$

Subject to

(43) $$\begin{array}{ll} g_{1}(x)= & 1.16-0.3717x_{2}x_{4}-0.00931x_{2}x_{10}-0.484x_{3}x_{9}\\ & +0.01343x_{6}x_{10}\leq 1 \end{array}$$

(44) $$\begin{array}{ll} g_{2}(x)= & 0.261-0.0159x_{1}x_{2}-0.188x_{1}x_{8}-0.019x_{2}x_{7}\\ & +0.0144x_{3}x_{5}+0.0008757x_{5}x_{10}+0.080405x_{6}x_{9}\\ & +0.00139x_{8}x_{11}+0.00001575x_{10}x_{11}\leq 0.32 \end{array}$$

(45) $$\begin{array}{ll} g_{3}(x)= & 0.214+0.00817x_{5}-0.131x_{1}x_{8}-0.0704x_{1}x_{9}\\ & +0.03099x_{2}x_{6}-0.018x_{2}x_{7}+0.0208x_{3}x_{8}+0.121x_{3}x_{9}\\ & -0.00364x_{5}x_{6}+0.0007715x_{5}x_{10}-0.000535x_{6}x_{10}\\ & +0.00121x_{8}x_{11}\leq 0.32 \end{array}$$

(46) $$\begin{array}{ll} g_{4}(x)= & 0.074-0.061x_{2}-0.163x_{3}x_{8}+0.001232x_{3}x_{10}\\ & -0.166x_{7}x_{9}+0.227x_{2}^{2}\leq 0.32 \end{array}$$

(47) $$\begin{array}{ll} g_{5}(x)= & 28.98+3.818x_{3}-4.2x_{1}x_{2}+0.0207x_{5}x_{10}+6.63x_{6}x_{9}\\ & -7.7x_{7}x_{8}+0.32x_{9}x_{10}\leq 32 \end{array}$$

(48) $$\begin{array}{ll} g_{6}(x)= & 33.86+2.95x_{3}+0.1792x_{10}-5.057x_{1}x_{2}-11.0x_{2}x_{8}\\ & -0.0215x_{5}x_{10}-9.98x_{7}x_{8}+22.0x_{8}x_{9}\leq 32 \end{array}$$

(49) $$g_{7}(x)=46.36-9.9x_{2}-12.9x_{1}x_{8}+0.1107x_{3}x_{10}\leq 32$$

(50) $$\begin{array}{ll} g_{8}(x)= & 4.72-0.5x_{4}-0.19x_{2}x_{3}-0.0122x_{4}x_{10}\\ & +0.009325x_{6}x_{10}+0.000191x_{11}^{2}\leq 4 \end{array}$$

(51) $$\begin{array}{ll} g_{9}(x)= & 10.58-0.674x_{1}x_{2}-1.95x_{2}x_{8}+0.02054x_{3}x_{10}\\ & -0.0198x_{4}x_{10}+0.028x_{6}x_{10}\leq 9.9 \end{array}$$

(52) $$\begin{array}{ll} g_{10}(x)= & 16.45-0.489x_{3}x_{7}-0.843x_{5}x_{6}+0.0432x_{9}x_{10}\\ & -0.0556x_{9}x_{11}-0.000786x_{11}^{2}\leq 15.7 \end{array}$$

Variable range

(53) $$0.5\leq x_{1}-x_{7}\leq 1.5,\;\;\;\;\;\;x_{8},x_{9}\in (0.192,0.345),\;\;\;\;\;\;-30\leq x_{10},x_{11}\leq 30$$

Table 7 outlines the contrastive results of the car side impact design. The GCRA did not require complex dimensional decoupling to update positions, which used relative position adjustment between individual and population optimal solutions to achieve optimization, naturally handling multi-parameter coupling relationships. The nonlinear control strategy provided dynamic feedback regulation to suppress fluctuations and quickly switch between GCRA and SCA to enhance exploration or development when the SCGCRA deviated from the optimal region. The SCGCRA possessed strong scalability and adaptability, which reduced the sensitivity of control parameters, promoted population diffusion, avoided premature convergence, focused on the optimal region, and eschewed search disorder. The global extremum solution is materialized by the SCGCRA at quantitative metrics: 0.5, 1.11643, 0.5, 1.30208, 0.5, 1.5, 0.5, 0.345, 0.192, −19.54935, and −0.00431, with the optimum weight of 22.84294.

### 5.5. Multiple-Disk Clutch Brake Design

**Figure 11:**
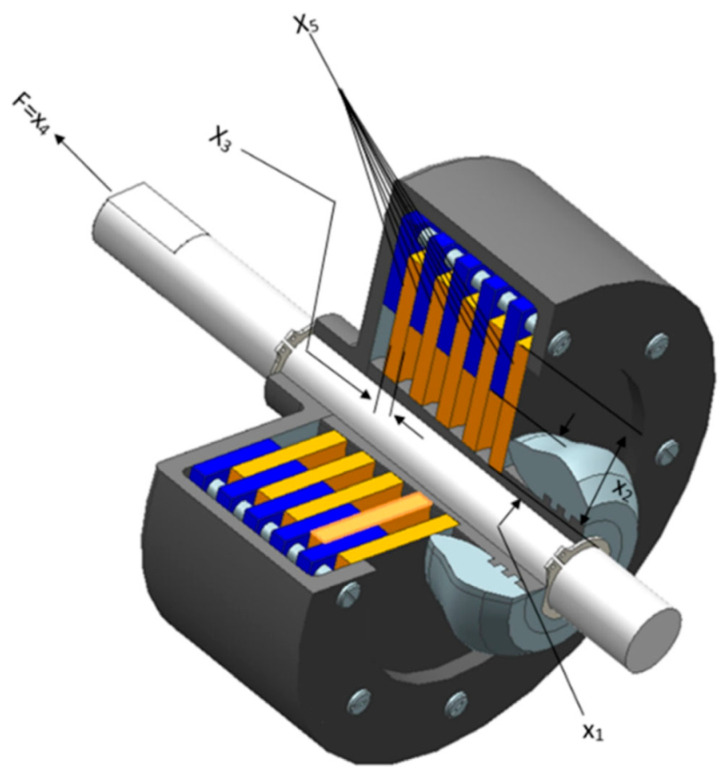
Sketch map of the multiple-disk clutch brake design.

The dominant motivation is to weaken (attenuate) the cumulative weight, which incorporates five quantitative metrics: thickness of disks (t), inner radius ($r_{i}$), outer radius ($r_{o}$), actuating force (F), and the number of friction surfaces (Z). Figure 11 portrays the sketch map of the multiple-disk clutch brake.

Consider

(54) $$x=[x_{1}\;\;\;x_{2}\;\;\;x_{3}\;\;\;x_{4}\;\;\;x_{5}]=[r_{i}\;\;\;r_{0}\;\;\;t\;\;\;F\;\;\;Z]$$

Minimize

(55) $$f(x)=\pi t\rho (r_{0}^{2}-r_{i}^{2})(Z+1)$$

Subject to

(56) $$g_{1}(x)=r_{0}-r_{i}-\Delta r\geq 0$$

(57) $$g_{2}(x)=l_{\max}-(Z+1)(t+\delta )\geq 0$$

(58) $$g_{3}(x)=p_{\max}+p_{rz}\geq 0$$

(59) $$g_{4}(x)=p_{\max}v_{sr\;\max}-p_{rz}v_{sr}\geq 0$$

(60) $$g_{5}(x)=v_{sr\;\max}-v_{sr}\geq 0$$

(61) $$g_{6}(x)=T_{\max}-T\geq 0$$

(62) $$g_{7}(x)=M_{h}-sM_{s}\geq 0$$

(63) $$g_{8}(x)=T\geq 0$$

(64) $$M_{h}=\frac{2}{3}\mu FZ\frac{r_{0}^{3}-r_{i}^{3}}{r_{0}^{2}-r_{i}^{2}}$$

(65) $$p_{rz}=\frac{F}{\pi (r_{0}^{2}-r_{i}^{2})}$$

(66) $$v_{sr}=\frac{2\pi n(r_{0}^{3}-r_{i}^{3})}{90(r_{0}^{2}-r_{i}^{2})}$$

(67) $$T=\frac{I_{z}\pi n}{30(M_{h}+M_{f})}$$

(68) $$\Delta r=20\;mm,\;\;\;I_{z}=55\;kgmm^{2},\;\;\;p_{\max}=1\;Mpa,\;\;\;F_{\max}=1000\;N$$

(69) $$T_{\max}=15\;s,\;\;\;\mu =0.5,\;\;\;s=1.5,\;\;\;M_{s}=40\;Nm$$

(70) $$M_{f}=3\;Nm,\;\;\;n=250\;rpm$$

(71) $$v_{sr\;\max}=10\;m/s,\;\;\;l_{\max}=30\;mm,\;\;\;r_{i\;\;\min}=60$$

(72) $$r_{i\;\max}=80,\;\;\;r_{o\;\min}=90$$

(73) $$r_{o\;\max}=110,\;\;\;t_{\min}=1.5,\;\;\;t_{\max}=3,\;\;\;F_{\min}=600$$

(74) $$F_{\max}=1000,\;\;\;Z_{\min}=2,\;\;\;Z_{\max}=9$$

Table 8 outlines the contrastive results of the multiple-disk clutch brake design. The SCA possesses a strong fine-tuning ability, ensuring the precise matching of control parameters with nonlinear system characteristics, thereby meeting the dual requirements of fast response and minimal system overshoot. The SCGCRA achieved closed-loop coordination between a nonlinear control strategy and GCRA optimization, which received control parameter feedback, thereby enhancing the robustness of the control system. The SCGCRA utilized the global coarse exploration and the local refined exploitation to promote search efficiency and flexibility, enhance convergence speed and solution accuracy, avoid search stagnation and dimensional disaster, strengthen adaptability and operability, and facilitate dynamic regulation and solution quality. The SCGCRA materialized the global extremum solution at quantitative metrics: 70, 90, 1, 600, and 2, with the optimum weight of 0.235247.

### 5.6. Rolling Element Bearing Design

**Figure 12:**
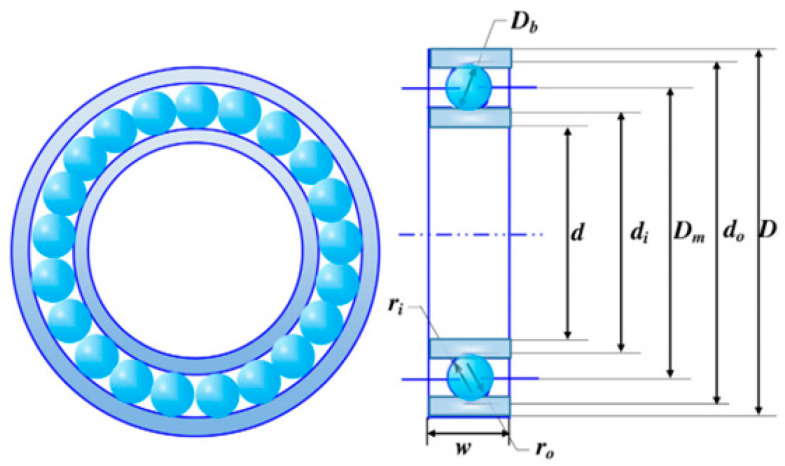
Sketch map of the rolling element bearing design.

The dominant motivation was to maximize the dynamic load-bearing capacity and optimum cost, which incorporates 10 quantitative metrics: pitch diameter ($D_{m}$), ball diameter ($D_{b}$), number of balls (Z), inner ($f_{i}$), and outer ($f_{o}$) raceway curvature coefficients, $K_{D\min}$, $K_{D\max}$, ε, e, and ζ. Figure 12 portrays the sketch map of the rolling element bearing design.

Consider

(75) $$\begin{array}{ll} x & =[x_{1}\;\;\;x_{2}\;\;\;x_{3}\;\;\;x_{4}\;\;\;x_{5}\;\;\;x_{6}\;\;\;x_{7}\;\;\;x_{8}\;\;\;x_{9}\;\;\;x_{10}]\\ & =[D_{m}\;D_{b}\;Z\;f_{i}\;f_{o}\;K_{D\min}\;K_{D\max}\;\varepsilon \;e\;\zeta ] \end{array}$$

Minimize

(76) $$C_{d}=\left\{\begin{array}{l} f_{c}Z^{2/3}D_{b}^{1.8},\;\;\;\;\;\;\;\;\;\;\;\;\;\;\;if\;\;D\leq 25.4mm\\ 3.647f_{c}Z^{2/3}D_{b}^{1.4},\;\;\;\;\;\;\;if\;\;\;D>25.4mm \end{array}\right.$$

Subject to

(77) $$g_{1}(x)=\frac{\phi_{0}}{2\sin^{-1}(D_{b}/D_{m})}-Z+1\leq 0$$

(78) $$g_{2}(x)=2D_{b}-K_{D\min}(D-d)\geq 0$$

(79) $$g_{3}(x)=K_{D\max}(D-d)-2D_{b}\geq 0$$

(80) $$g_{4}(x)=\zeta B_{\omega }-D_{b}\leq 0$$

(81) $$g_{5}(x)=D_{m}-0.5(D+d)\geq 0$$

(82) $$g_{6}(x)=(0.5+e)(D+d)-D_{m}\geq 0$$

(83) $$g_{7}(x)=0.5(D-D_{m}-D_{b})-\varepsilon D_{b}\geq 0$$

(84) $$g_{8}(x)=f_{i}\geq 0.515$$

(85) $$g_{9}(x)=f_{o}\geq 0.515$$

(86) fc=37.911+1.041−r1+r1.72fi(2fo−1)fo(2fi−1)0.41103−0.3r0.3(1−r)1.39(1+r)1/32fi2fi−10.41

(87) $$x={\left\{\frac{\left(D-d\right)}{2}-3\frac{T}{4}\right\}}^{2}+{\left\{\frac{D}{2}-\frac{T}{4}-D_{b}\right\}}^{2}-{\left\{\frac{d}{2}+\frac{T}{4}\right\}}^{2}$$

(88) $$y=2\left\{\frac{\left(D-d\right)}{2}-3\frac{T}{4}\right\}\left\{\frac{D}{2}-\frac{T}{4}-D_{b}\right\}$$

(89) $$\phi_{o}=2\pi -2\cos^{-1}\left(\frac{x}{y}\right)$$

(90) $$r=\frac{D_{b}}{D_{m}},\;\;\;f_{i}=\frac{r_{i}}{D_{b}},\;\;\;f_{o}=\frac{r_{o}}{D_{b}},\;\;\;T=D-d-2D_{b}$$

(91) $$D=160,\;\;\;d=90,\;\;\;B_{\omega }=30,\;\;\;r_{i}=r_{o}=11.033$$

Variable range

(92) $$0.5(D+d)\leq D_{m}\leq 0.6(D+d)$$

(93) $$0.15(D-d)\leq D_{b}\leq 0.45(D-d)$$

(94) $$4\leq Z\leq 50,\;\;\;0.515\leq f_{i},f_{o}\leq 0.6$$

(95) $$0.4\leq K_{D\min}\leq 0.5,\;\;\;0.6\leq K_{D\min}\leq 0.7$$

(96) 0.3≤ε≤0.4,   0.02≤e≤0.1,   0.6≤ζ≤0.85

Table 9 outlines the contrastive results of the rolling element bearing design. The GCRA and SCA both contain control parameters that need to be set. The introduction of a nonlinear control strategy compensated for the performance loss caused by suboptimal sub-algorithm parameters through its adaptive scheduling mechanism. The nonlinear control strategy dynamically adjusted the dominance of GCRA and SCA based on real-time feedback signals such as iteration progress, population diversity, or convergence speed. The SCGCRA utilized the SCA’s mathematical periodic oscillatory fluctuation characteristics to overlap the global exploration scope, locate potential optimal areas, and enhance population diversity. The SCGCRA employed a nonlinear control strategy to adjust dispersion, minimize ineffective exploration, and improve adaptability and complementarity. The global extremum solution was materialized by the SCGCRA at the following quantitative metrics: 126.2339, 20.1947, 10.5139, 0.5524, 0.5428, 0.4072, 0.6565, 0.3254, 0.0681, and 0.6142, with an optimum cost of 90,020.39.

## 6. Conclusions and Future Works

This paper portrays the SCGCRA to address the twenty-three benchmark functions and six constrained real-world engineering designs; the dominant motivation is to balance global coarse exploration and local refined exploitation to identify the superior quantitative metrics, detection efficiency, and solution accuracy, and exhibit strong practicality and adaptability to strengthen solution quality and discover the global extremum or high-quality approximate solution. To address the GCRA’s serious drawbacks of the high parameter sensitivity, insufficient solution accuracy, high computational complexity, susceptibility to local optima and overfitting, poor dynamic adaptability, and severe curse of dimensionality, the SCGCRA combines the periodic oscillatory fluctuation characteristics of the SCA and the anti-disturbance ability and nonlinear processing characteristics of the nonlinear control strategy to realize repeated expansion and contraction, facilitate dynamic regulation and population diversity, avert dimensional disaster and search oscillation, achieve synergistic complementarity and reduce sensitivity, and quantify the solution accuracy and search efficiency. The SCGCRA possesses strong flexibility and operability, enabling multi-directional perturbation paths that achieve high-precision local convergence, narrow the search solution space, expand the global exploration scope, locate potential optimal areas, strengthen local exploitation, and enhance solution accuracy. The SCGCRA is compared with the CPO, BKA, EHO, PO, WSA, HLOA, ECO, IAO, AO, HEOA, NRBO, and GCRA. The experimental results demonstrate that the SCGCRA exhibits substantial superiority and responsibility in remedying the disequilibrium between exploration and exploitation, thereby accelerating convergence speed, enhancing solution accuracy, and attaining the global extremum solution.

In future research, we will utilize the SCGCRA to resolve the numerical experiments of CEC2017, CEC2019, CEC2020, CEC2021, and CEC2022, which will further verify the practicality and reliability of the proposed algorithm. We will utilize the maximum fitness evaluations to compare the performance of different algorithms fairly. We will leverage the Anhui Provincial Engineering Research Center for Intelligent Equipment for Under-forest Crops to showcase the core aspects: deep technological integration and innovation, breakthroughs in equipment for specialty crops, and the upgrading of green and intelligent equipment. We will combine the distributed computing capabilities of the SCGCRA with the decentralized characteristics of under-forest crops to achieve thoughtful and efficient detection, and bionic precision harvesting equipment, thereby reducing redundant searches and enhancing detection efficiency. We will utilize the dynamic task allocation capability and optimization of tillage depth and frequency provided by the SCGCRA to exploit lightweight and modular equipment that is detachable and easy to transport, thereby reducing soil damage. We will utilize the energy management strategy of the SCGCRA to develop low-power, long-endurance harvesting robots, reduce carbon emissions, achieve variable fertilization and precise irrigation, dwindle resource waste, and promote the coordinated progress of agriculture and ecological protection.

## Figures and Tables

**Table 1:** Benchmark functions.

Benchmark Test Functions	Dim	Range	$f_{\min}$
$f_{1}=\sum_{i=1}^{n}x_{i}^{2}$	30	[−100, 100]	0
$f_{2}(x)=\sum_{i=1}^{n}\left|x_{i}\right|+\prod_{i=1}^{n}\left|x_{i}\right|$	30	[−10, 10]	0
$f_{3}(x)=\sum_{i=1}^{n}(\sum_{j=1}^{i}x_{j}{)}^{2}$	30	[−100, 100]	0
$f_{4}(x)=\max_{i}\{|x_{i}|,1\leq i\leq n\}$	30	[−100, 100]	0
$f_{5}(x)=\sum_{i=1}^{n-1}\left[100{\left(x_{i+1}-x_{i}^{2}\right)}^{2}+{\left(x_{i}-1\right)}^{2}\right]$	30	[−30, 30]	0
$f_{6}(x)=\sum_{i=1}^{n}{\left([x_{i}+0.5]\right)}^{2}$	30	[−100, 100]	0
$f_{7}(x)=\sum_{i=1}^{n}ix_{i}^{4}+random[0,1)$	30	[−1.28, 1.28]	0
$f_{8}(x)=\sum_{i=1}^{n}\left[x_{i}^{2}-10\cos(2\pi x_{i})+10\right]$	30	[−5.12, 5.12]	0
$\begin{array}{l} f_{9}(x)=-20\exp\left(-0.2\sqrt{\frac{1}{n}\sum_{i=1}^{n}x_{i}^{2}}-\exp\left(\frac{1}{n}\sum_{i=1}^{n}\cos2\pi x_{i}\right)\right)\\ +20+e \end{array}$	30	[−32, 32]	0
$f_{10}(x)=\frac{1}{4000}\sum_{i=1}^{n}\left(x_{i}^{2}\right)-\prod_{i=1}^{n}\cos\left(\frac{x_{i}}{\sqrt{i}}\right)+1$	30	[−600, 600]	0
$\begin{array}{l} f_{11}(x)=\frac{\pi }{n}\left\{10\sin^{2}(\pi y_{1})+\sum_{i=1}^{n-1}\begin{array}{l} {\left(y-1\right)}^{2}[1+10\sin^{2}(\pi y_{1})]\\ +{\left(y_{n}-1\right)}^{2} \end{array}\right\}\\ +\sum_{i=1}^{n}u(x_{i},10,100,4)\\ y_{i}=1+\frac{x_{i}+1}{4}\\ u(x_{i},a,k,m)=\left\{\begin{array}{l} k{\left(x_{i}-a\right)}^{m},x^{i}>a\\ 0,-a\leq x_{i}\leq a\\ k{\left(-x_{i}-z\right)}^{m},x_{i}<a \end{array}\right. \end{array}$	30	[−50, 50]	0
$\begin{array}{l} f_{12}(x)=0.1\left\{\sin^{2}\left(3\pi x_{1}+\sum_{i=1}^{n}\begin{array}{l} {\left(x_{i}-1\right)}^{2}[1+\sin^{2}(3\pi x_{i}+1)]\\ +{\left(x_{n}-1\right)}^{2}[1+\sin^{2}(2\pi x_{n})] \end{array}\right)\right\}\\ +\sum_{i=1}^{n}u(x_{i},5,100,4) \end{array}$	30	[−50, 50]	0
$f_{13}(x)=(\frac{1}{500}+\sum_{j=1}^{25}\frac{1}{j+\sum_{i=1}^{2}{\left(x_{i}-a_{ij}\right)}^{6}}{)}^{-1}$	2	[−65, 65]	0.998
$f_{14}(x)=4x_{1}^{2}-2.1x_{1}^{4}+\frac{1}{3}x_{1}^{6}+x_{1}x_{2}-4x_{2}^{2}+4x_{2}^{4}$	2	[−5, 5]	−1.0316
$f_{15}(x)=-\frac{1+\cos(12\sqrt{x_{1}^{2}+x_{2}^{2}})}{0.5(x_{1}^{2}+x_{2}^{2})+2}$	2	[−5.12, 5.12]	−1
$\begin{array}{l} f_{16}(x)=[1+{\left(x_{1}+x_{2}+1\right)}^{2}\left(19-14x_{1}+3x_{1}^{2}-14x_{2}+6x_{1}x_{2}+3x_{2}^{2}\right)]\\ \;\times \;[30+{\left(2x_{1}-3x_{2}\right)}^{2}(18-32x_{1}+12x_{1}^{2}+48x_{2}-36x_{1}x_{2}+27x_{2}^{2})] \end{array}$	2	[−2, 2]	3
$f_{17}(x)=-\sum_{i=1}^{4}c_{i}\exp(-\sum_{j=1}^{6}a_{ij}{\left(x_{j}-p_{ij}\right)}^{2})$	6	[0, 1]	−3.32
$f_{18}(x)=-\sum_{i=1}^{5}{\left[(x-a_{i}){\left(x-a_{i}\right)}^{T}+c_{i}\right]}^{-1}$	4	[0, 10]	−10.1532
$f_{19}(x)=-\sum_{i=1}^{7}{\left[(x-a_{i}){\left(x-a_{i}\right)}^{T}+c_{i}\right]}^{-1}$	4	[0, 10]	−10.4029
$f_{20}(x)=-\sum_{i=1}^{10}{\left[(x-a_{i}){\left(x-a_{i}\right)}^{T}+c_{i}\right]}^{-1}$	4	[0, 10]	−10.5364
$f_{21}(x)=-\cos(x_{1})\cos(x_{2})\exp(-{\left(x_{1}-\pi \right)}^{2}-{\left(x_{2}-\pi \right)}^{2})$	2	[−2π,2π]	−1
$f_{22}(x)=0.5+\frac{\sin^{2}\left(\sqrt{x_{1}^{2}+x_{2}^{2}}\right)-0.5}{{\left(1+0.001(x_{1}^{2}+x_{2}^{2})\right)}^{2}}$	2	[−100, 100]	−1
$f_{23}(x)=\sum_{i=1}^{n}x_{i}\sin(x_{i})+0.1x_{i}$	10	[−10, 10]	0

**Table 2:** Contrastive results of benchmark functions.

**Function**	**Result**	**CPO**	**BKA**	**EHO**	**PO**	**WSA**	HLOA	ECO	IAO	AO	HEOA	NRBO	GCRA	SCGCRA
$f_{1}$	Best	$3.9\;\times \;10^{-191}$	$7.4\;\times \;10^{-221}$	$7.22\;\times \;10^{-22}$	0	0	0	$8.3\;\times \;10^{-177}$	0	$5.86\;\times \;10^{-51}$	$3.5\;\times \;10^{-188}$	0	0	0
	Worst	$1.2\;\times \;10^{-135}$	$8.2\;\times \;10^{-181}$	$1.03\;\times \;10^{-18}$	0	0	0	$2.5\;\times \;10^{-104}$	0	$1.26\;\times \;10^{-36}$	$3.1\;\times \;10^{-154}$	0	0	0
	Mean	$3.9\;\times \;10^{-137}$	$2.7\;\times \;10^{-182}$	$1.05\;\times \;10^{-19}$	0	0	0	$8.4\;\times \;10^{-106}$	0	$7.51\;\times \;10^{-38}$	$1.0\;\times \;10^{-155}$	0	0	0
	Std	$2.1\;\times \;10^{-136}$	0	$2.50\;\times \;10^{-19}$	0	0	0	$4.6\;\times \;10^{-105}$	0	$2.62\;\times \;10^{-37}$	$5.7\;\times \;10^{-155}$	0	0	0
$f_{2}$	Best	$1.40\;\times \;10^{-99}$	$6.5\;\times \;10^{-109}$	$2.33\;\times \;10^{-14}$	$9.5\;\times \;10^{-274}$	$7.4\;\times \;10^{-157}$	$1.3\;\times \;10^{-270}$	$1.14\;\times \;10^{-89}$	0	$1.41\;\times \;10^{-36}$	$1.84\;\times \;10^{-93}$	0	0	0
	Worst	$1.48\;\times \;10^{-70}$	$4.71\;\times \;10^{-94}$	$5.06\;\times \;10^{-11}$	$2.2\;\times \;10^{-265}$	$5.2\;\times \;10^{-151}$	$1.4\;\times \;10^{-251}$	$1.41\;\times \;10^{-52}$	0	$8.28\;\times \;10^{-26}$	$4.30\;\times \;10^{-69}$	$1.6\;\times \;10^{-306}$	0	0
	Mean	$4.92\;\times \;10^{-72}$	$1.60\;\times \;10^{-95}$	$2.41\;\times \;10^{-12}$	$1.5\;\times \;10^{-266}$	$2.4\;\times \;10^{-152}$	$5.9\;\times \;10^{-253}$	$4.75\;\times \;10^{-54}$	0	$7.50\;\times \;10^{-27}$	$1.44\;\times \;10^{-70}$	$8.9\;\times \;10^{-308}$	0	0
	Std	$2.69\;\times \;10^{-71}$	$8.95\;\times \;10^{-95}$	$9.19\;\times \;10^{-12}$	0	$9.6\;\times \;10^{-152}$	0	$2.57\;\times \;10^{-53}$	0	$1.82\;\times \;10^{-26}$	$7.85\;\times \;10^{-70}$	0	0	0
$f_{3}$	Best	$9.5\;\times \;10^{-186}$	$4.7\;\times \;10^{-219}$	1.719617	0	0	0	$5.1\;\times \;10^{-165}$	0	$3.60\;\times \;10^{-17}$	$1.6\;\times \;10^{-216}$	0	0	0
	Worst	$1.9\;\times \;10^{-142}$	$5.6\;\times \;10^{-158}$	23.04144	0	$4.0\;\times \;10^{-307}$	0	$4.0\;\times \;10^{-115}$	0	0.000685	$4.9\;\times \;10^{-205}$	0	0	0
	Mean	$7.7\;\times \;10^{-144}$	$1.9\;\times \;10^{-159}$	9.040724	0	0	0	$1.3\;\times \;10^{-116}$	0	$5.04\;\times \;10^{-05}$	$1.6\;\times \;10^{-206}$	0	0	0
	Std	$3.5\;\times \;10^{-143}$	$1.0\;\times \;10^{-158}$	5.778427	0	0	0	$7.4\;\times \;10^{-116}$	0	0.000139	0	0	0	0
$f_{4}$	Best	$1.60\;\times \;10^{-94}$	$5.3\;\times \;10^{-109}$	4.708022	$5.9\;\times \;10^{-277}$	$4.1\;\times \;10^{-164}$	$1.2\;\times \;10^{-281}$	$1.68\;\times \;10^{-82}$	0	$3.46\;\times \;10^{-07}$	$1.19\;\times \;10^{-58}$	0	0	0
	Worst	$5.40\;\times \;10^{-73}$	$9.62\;\times \;10^{-94}$	14.89710	$4.3\;\times \;10^{-269}$	$1.3\;\times \;10^{-152}$	$4.2\;\times \;10^{-250}$	$2.93\;\times \;10^{-58}$	0	$7.22\;\times \;10^{-05}$	$1.74\;\times \;10^{-48}$	$5.9\;\times \;10^{-296}$	0	0
	Mean	$1.94\;\times \;10^{-74}$	$3.21\;\times \;10^{-95}$	9.530409	$1.8\;\times \;10^{-270}$	$4.4\;\times \;10^{-154}$	$1.6\;\times \;10^{-251}$	$1.05\;\times \;10^{-59}$	0	$2.44\;\times \;10^{-05}$	$7.96\;\times \;10^{-50}$	$2.1\;\times \;10^{-297}$	0	0
	Std	$9.86\;\times \;10^{-74}$	$1.76\;\times \;10^{-94}$	2.750975	0	$2.4\;\times \;10^{-153}$	0	$5.35\;\times \;10^{-59}$	0	$1.87\;\times \;10^{-05}$	$3.31\;\times \;10^{-49}$	0	0	0
$f_{5}$	Best	28.22641	23.97181	2.096586	$8.62\;\times \;10^{-05}$	16.70165	$3.36\;\times \;10^{-06}$	25.55554	28.64800	26.33154	0.128208	26.62669	$1.03\;\times \;10^{-08}$	$4.20\;\times \;10^{-12}$
	Worst	28.97101	28.91524	152.7259	25.71341	20.93888	28.70570	26.46565	28.77166	26.80314	1.643262	28.81900	0.000118	$6.95\;\times \;10^{-06}$
	Mean	28.80065	26.19458	52.40917	23.79129	18.89737	20.09465	25.94569	28.72375	26.60947	0.589470	27.49856	$9.89\;\times \;10^{-06}$	$8.75\;\times \;10^{-07}$
	Std	0.146114	1.377809	39.41412	4.524737	1.226874	13.35128	0.258348	0.026958	0.126309	0.381245	0.603713	$2.40\;\times \;10^{-05}$	$1.59\;\times \;10^{-06}$
$f_{6}$	Best	6.032364	$3.93\;\times \;10^{-05}$	$8.63\;\times \;10^{-23}$	$1.93\;\times \;10^{-10}$	0	$3.77\;\times \;10^{-06}$	$1.57\;\times \;10^{-10}$	0.170839	$2.35\;\times \;10^{-10}$	0.001802	1.564431	$2.03\;\times \;10^{-10}$	$4.13\;\times \;10^{-13}$
	Worst	6.750920	5.838999	$7.70\;\times \;10^{-19}$	$7.70\;\times \;10^{-09}$	0	0.000167	$4.97\;\times \;10^{-07}$	1.827244	$5.58\;\times \;10^{-05}$	1.483572	3.069441	$2.93\;\times \;10^{-07}$	$7.77\;\times \;10^{-09}$
	Mean	6.493847	0.712540	$6.86\;\times \;10^{-20}$	$1.73\;\times \;10^{-09}$	0	$6.09\;\times \;10^{-05}$	$6.50\;\times \;10^{-08}$	0.946642	$5.18\;\times \;10^{-06}$	0.257351	2.262051	$5.62\;\times \;10^{-08}$	$1.04\;\times \;10^{-09}$
	Std	0.183935	1.678794	$1.56\;\times \;10^{-19}$	$1.93\;\times \;10^{-09}$	0	$4.49\;\times \;10^{-05}$	$1.11\;\times \;10^{-07}$	0.445780	$1.11\;\times \;10^{-05}$	0.421081	0.364347	$7.19\;\times \;10^{-08}$	$1.70\;\times \;10^{-09}$
$f_{7}$	Best	$5.29\;\times \;10^{-07}$	$1.85\;\times \;10^{-06}$	0.016694	$1.70\;\times \;10^{-06}$	$6.76\;\times \;10^{-07}$	$1.46\;\times \;10^{-06}$	$2.74\;\times \;10^{-06}$	$8.06\;\times \;10^{-07}$	0.000187	$1.59\;\times \;10^{-06}$	$3.42\;\times \;10^{-06}$	$8.72\;\times \;10^{-07}$	$1.48\;\times \;10^{-08}$
	Worst	0.000306	0.000312	0.154408	0.000199	0.000114	0.000396	0.000232	$6.07\;\times \;10^{-05}$	0.002812	0.000243	0.000379	0.000111	$2.02\;\times \;10^{-06}$
	Mean	$6.87\;\times \;10^{-05}$	$8.94\;\times \;10^{-05}$	0.064251	$6.74\;\times \;10^{-05}$	$3.95\;\times \;10^{-05}$	0.000124	$8.52\;\times \;10^{-05}$	$2.29\;\times \;10^{-05}$	0.001062	$4.96\;\times \;10^{-05}$	0.000112	$4.45\;\times \;10^{-05}$	$5.64\;\times \;10^{-07}$
	Std	$6.91\;\times \;10^{-05}$	$8.54\;\times \;10^{-05}$	0.034555	$5.00\;\times \;10^{-05}$	$3.17\;\times \;10^{-05}$	0.000104	$6.18\;\times \;10^{-05}$	$1.66\;\times \;10^{-05}$	0.000603	$4.66\;\times \;10^{-05}$	$9.89\;\times \;10^{-05}$	$3.15\;\times \;10^{-05}$	$5.11\;\times \;10^{-07}$
$f_{8}$	Best	0	0	16.91430	0	0	0	0	0	0	29.40100	0	0	0
	Worst	0	0	79.59648	0	0	0	0	0	0	38.57384	0	0	0
	Mean	0	0	27.82566	0	0	0	0	0	0	30.84521	0	0	0
	Std	0	0	10.91009	0	0	0	0	0	0	2.421543	0	0	0
$f_{9}$	Best	$4.44\;\times \;10^{-16}$	$4.44\;\times \;10^{-16}$	$5.87\;\times \;10^{-12}$	$4.44\;\times \;10^{-16}$	$4.44\;\times \;10^{-16}$	$4.44\;\times \;10^{-16}$	$4.44\;\times \;10^{-16}$	$4.44\;\times \;10^{-16}$	$4.00\;\times \;10^{-15}$	$4.44\;\times \;10^{-16}$	$4.44\;\times \;10^{-16}$	$4.44\;\times \;10^{-16}$	$4.44\;\times \;10^{-16}$
	Worst	$4.44\;\times \;10^{-16}$	$4.44\;\times \;10^{-16}$	3.681357	$4.44\;\times \;10^{-16}$	$4.44\;\times \;10^{-16}$	$4.44\;\times \;10^{-16}$	$4.44\;\times \;10^{-16}$	$4.44\;\times \;10^{-16}$	$1.47\;\times \;10^{-14}$	$4.44\;\times \;10^{-16}$	$4.44\;\times \;10^{-16}$	$4.44\;\times \;10^{-16}$	$4.44\;\times \;10^{-16}$
	Mean	$4.44\;\times \;10^{-16}$	$4.44\;\times \;10^{-16}$	1.863179	$4.44\;\times \;10^{-16}$	$4.44\;\times \;10^{-16}$	$4.44\;\times \;10^{-16}$	$4.44\;\times \;10^{-16}$	$4.44\;\times \;10^{-16}$	$8.85\;\times \;10^{-15}$	$4.44\;\times \;10^{-16}$	$4.44\;\times \;10^{-16}$	$4.44\;\times \;10^{-16}$	$4.44\;\times \;10^{-16}$
	Std	0	0	0.783795	0	0	0	0	0	$3.02\;\times \;10^{-15}$	0	0	0	0
$f_{10}$	Best	0	0	0	0	0	0	0	0	0	0	0	0	0
	Worst	0	0	0.157370	0	0	0	0	0	0.075727	0.082115	0	0	0
	Mean	0	0	0.021437	0	0	0	0	0	0.003756	0.022793	0	0	0
	Std	0	0	0.038474	0	0	0	0	0	0.014409	0.026793	0	0	0
$f_{11}$	Best	0.811592	$2.76\;\times \;10^{-06}$	$2.38\;\times \;10^{-22}$	$9.83\;\times \;10^{-12}$	$1.77\;\times \;10^{-30}$	$1.11\;\times \;10^{-08}$	$5.86\;\times \;10^{-11}$	0.018092	$3.48\;\times \;10^{-13}$	$1.81\;\times \;10^{-05}$	0.088837	$2.72\;\times \;10^{-13}$	$4.06\;\times \;10^{-13}$
	Worst	1.402394	0.292436	0.936925	$1.19\;\times \;10^{-09}$	0.207317	0.207367	$7.60\;\times \;10^{-08}$	0.231948	$9.63\;\times \;10^{-07}$	0.960863	0.350026	$8.59\;\times \;10^{-09}$	$3.05\;\times \;10^{-09}$
	Mean	0.999341	0.016193	0.214786	$1.37\;\times \;10^{-10}$	0.024187	0.006916	$4.73\;\times \;10^{-09}$	0.085527	$6.23\;\times \;10^{-08}$	0.199670	0.183719	$1.34\;\times \;10^{-09}$	$2.05\;\times \;10^{-10}$
	Std	0.156332	0.052893	0.326651	$2.31\;\times \;10^{-10}$	0.064897	0.037859	$1.38\;\times \;10^{-08}$	0.056073	$1.79\;\times \;10^{-07}$	0.275405	0.062374	$2.23\;\times \;10^{-09}$	$5.56\;\times \;10^{-10}$
$f_{12}$	Best	2.703748	0.206470	$5.47\;\times \;10^{-21}$	$1.62\;\times \;10^{-11}$	$3.96\;\times \;10^{-31}$	$4.56\;\times \;10^{-06}$	$1.07\;\times \;10^{-09}$	0.074259	$2.41\;\times \;10^{-12}$	0.000135	1.157680	$3.42\;\times \;10^{-10}$	$4.92\;\times \;10^{-13}$
	Worst	2.898933	2.996717	3.608452	$1.13\;\times \;10^{-08}$	0.030827	0.108559	0.801424	2.975089	0.586973	0.004159	2.791573	$2.30\;\times \;10^{-07}$	$1.27\;\times \;10^{-08}$
	Mean	2.803325	1.468360	0.620813	$1.39\;\times \;10^{-09}$	0.002795	0.009825	0.030724	2.167549	0.111098	0.001434	1.980314	$3.94\;\times \;10^{-08}$	$2.74\;\times \;10^{-09}$
	Std	0.042338	0.754096	1.358982	$2.24\;\times \;10^{-09}$	0.007703	0.025663	0.145685	1.257903	0.133287	0.001229	0.430654	$6.12\;\times \;10^{-08}$	$4.13\;\times \;10^{-09}$
$f_{13}$	Best	1.000194	0.998004	0.998004	0.998004	0.998004	0.998004	0.998004	0.998004	0.998004	0.998004	0.998004	0.998004	0.998004
	Worst	2.982105	1.992031	10.76318	0.998004	1.992031	12.67051	2.982105	0.998004	10.76318	12.67051	2.982105	0.998004	0.998004
	Mean	2.029939	1.031138	2.311901	0.998004	1.031138	4.888682	1.130277	0.998004	1.522078	7.734534	1.460961	0.998004	0.998004
	Std	0.756035	0.181484	2.323848	0	0.181484	3.849528	0.503383	0	1.828591	5.035198	0.853527	$1.78\;\times \;10^{-12}$	$1.33\;\times \;10^{-10}$
$f_{14}$	Best	−1.03160	−1.03163	−1.03163	−1.03163	−1.03163	−1.03163	−1.03163	−1.03163	−1.03163	−1.03163	−1.03163	−1.03161	−1.03163
	Worst	−1.02996	−1.03163	−1.03163	−1.03163	−1.03163	−0.21546	−1.03163	−1.03163	−1.03141	−1.03125	−1.03163	−0.83077	−1.03163
	Mean	−1.03127	−1.03163	−1.03163	−1.03163	−1.03163	−1.00442	−1.03163	−1.03163	−1.03161	−1.03156	−1.03163	−0.99581	−1.03163
	Std	0.000390	$5.90\;\times \;10^{-16}$	$6.78\;\times \;10^{-16}$	$6.78\;\times \;10^{-16}$	$6.52\;\times \;10^{-16}$	0.149011	$3.40\;\times \;10^{-15}$	$6.78\;\times \;10^{-16}$	$5.39\;\times \;10^{-05}$	$8.67\;\times \;10^{-05}$	$5.83\;\times \;10^{-16}$	0.044594	$6.58\;\times \;10^{-16}$
$f_{15}$	Best	−1	−1	−1	−1	−1	−1	−1	−1	−1	−1	−1	−1	−1
	Worst	−1	−1	−0.93625	−0.93625	−1	−1	−1	−1	−1	−1	−1	−1	−1
	Mean	−1	−1	−0.98725	−0.99787	−1	−1	−1	−1	−1	−1	−1	−1	−1
	Std	0	0	0.025938	0.011640	0	0	0	0	0	0	0	$4.42\;\times \;10^{-12}$	0
$f_{16}$	Best	3	3	3	3	3	3	3	3	3	3.000011	3	3.129500	3
	Worst	3.000200	3	3	3	3	30	3	3	3.044026	3.029225	3	32.68454	3
	Mean	3.000027	3	3	3	3	3.9	3	3	3.003641	3.003765	3	18.45967	3
	Std	$4.37\;\times \;10^{-05}$	$7.14\;\times \;10^{-16}$	$1.75\;\times \;10^{-15}$	$1.10\;\times \;10^{-15}$	$9.72\;\times \;10^{-16}$	4.929503	$1.10\;\times \;10^{-15}$	$1.82\;\times \;10^{-15}$	0.008607	0.006171	$3.77\;\times \;10^{-15}$	12.04699	$1.66\;\times \;10^{-15}$
$f_{17}$	Best	−3.15823	−3.32200	−3.32200	−3.32200	−3.32200	−3.32200	−3.32200	−3.32200	−3.32200	−3.30996	−3.32200	−2.60843	−3.32200
	Worst	−2.45031	−3.15869	−3.20310	−3.20310	−3.20310	−3.08394	−3.20310	−3.32200	−3.20166	−3.06329	−3.13725	−1.16984	−3.32200
	Mean	−2.94344	−3.29920	−3.29029	−3.29029	−3.25066	−3.25655	−3.25462	−3.32200	−3.27040	−3.19238	−3.25994	−1.82424	−3.32200
	Std	0.144663	0.052318	0.053475	0.053475	0.059241	0.075305	0.059923	$1.36\;\times \;10^{-15}$	0.060001	0.072875	0.067223	0.391285	$9.58\;\times \;10^{-15}$
$f_{18}$	Best	−4.06428	−10.1532	−10.1532	−10.1532	−10.1532	−10.1532	−10.1532	−10.1532	−10.1532	−10.1520	−10.1532	−10.1532	−10.1532
	Worst	−0.85099	−10.1532	−2.63047	−10.1532	−5.05520	−2.63047	−2.63047	−5.05520	−2.62952	−7.07049	−5.38758	−10.1494	−10.1521
	Mean	−1.58082	−10.1532	−8.31182	−10.1532	−9.64340	−9.65092	−9.90244	−7.43427	−5.85794	−9.16726	−9.98192	−10.1528	−10.1531
	Std	0.975053	$5.71\;\times \;10^{-15}$	2.938369	$6.51\;\times \;10^{-15}$	1.555546	1.908370	1.373456	2.586809	2.813123	0.870257	0.869491	0.000723	0.000198
$f_{19}$	Best	−3.87932	−10.4029	−10.4029	−10.4029	−10.4029	−10.4029	−10.4029	−10.4029	−10.4029	−10.4005	−10.4029	−10.4028	−10.4028
	Worst	−0.84842	−3.72430	−2.75193	−2.76590	−5.08767	−2.76590	−1.83759	−5.08767	−2.74761	−6.51320	−4.89939	−10.3973	−10.4020
	Mean	−2.01752	−10.1794	−9.41615	−10.1484	−9.33989	−8.71556	−9.16305	−7.56813	−6.89784	−9.30859	−9.95802	−10.4024	−10.4027
	Std	0.919006	1.219188	2.563526	1.394327	2.162454	3.116658	2.835141	2.697054	3.610540	1.122435	1.405488	0.001098	0.000190
$f_{20}$	Best	−4.18195	−10.5364	−10.5364	−10.5364	−10.5364	−10.5364	−10.5364	−10.5364	−10.5364	−10.5339	−10.5364	−10.5363	−10.5363
	Worst	−0.93680	−10.5364	−2.87114	−3.83543	−5.12848	−2.42173	−2.42173	−5.12848	−2.42144	−8.11593	−7.26135	−10.5341	−10.5359
	Mean	−2.66391	−10.5364	−9.83417	−10.0897	−9.81535	−8.46676	−8.74623	−6.75086	−6.70094	−9.84772	−10.2548	−10.5360	−10.5362
	Std	0.816296	$2.03\;\times \;10^{-15}$	2.147720	1.700094	1.869769	3.504272	3.332191	2.520590	3.740067	0.780452	0.749936	0.000594	$9.39\;\times \;10^{-05}$
$f_{21}$	Best	−0.99988	−1	−1	−1	−1	−1	−1	−1	−1	−1	−1	−1	−1
	Worst	−0.93514	−1	−1	−1	−1	−1	−1	−1	−0.99901	−0.99996	−1	−1	−1
	Mean	−0.98330	−1	−1	−1	−1	−1	−1	−1	−0.99992	−0.99999	−1	−1	−1
	Std	0.014053	0	0	0	0	0	0	0	0.000223	$9.32\;\times \;10^{-06}$	0	$8.37\;\times \;10^{-08}$	$2.55\;\times \;10^{-07}$
$f_{22}$	Best	−1	−1	−1	−1	−1	−1	−1	−1	−1	−1	−1	−1	−1
	Worst	−1	−1	−0.99028	−0.99028	−1	−1	−1	−1	−1	−0.99028	−1	−1	−1
	Mean	−1	−1	−0.99255	−0.99870	−1	−1	−1	−1	−1	−0.99636	−1	−1	−1
	Std	0	0	0.004180	0.003359	0	0	0	0	0	0.004718	0	$4.41\;\times \;10^{-10}$	0
$f_{23}$	Best	$6.4\;\times \;10^{-140}$	$1.1\;\times \;10^{-110}$	$5.52\;\times \;10^{-97}$	$2.7\;\times \;10^{-275}$	$5.5\;\times \;10^{-181}$	0	$1.39\;\times \;10^{-80}$	0	$1.17\;\times \;10^{-87}$	8.05 × 10^−97^	0	$4.08\;\times \;10^{-14}$	0
	Worst	$2.4\;\times \;10^{-111}$	$9.07\;\times \;10^{-87}$	$1.44\;\times \;10^{-15}$	$4.2\;\times \;10^{-263}$	$7.43\;\times \;10^{-05}$	$1.6\;\times \;10^{-250}$	$1.33\;\times \;10^{-61}$	0	$9.34\;\times \;10^{-51}$	0.378388	0	$1.29\;\times \;10^{-05}$	0
	Mean	$8.1\;\times \;10^{-113}$	$3.02\;\times \;10^{-88}$	$1.31\;\times \;10^{-16}$	$3.6\;\times \;10^{-264}$	$6.41\;\times \;10^{-06}$	$5.3\;\times \;10^{-252}$	$8.56\;\times \;10^{-63}$	0	$3.19\;\times \;10^{-52}$	0.030604	0	$6.22\;\times \;10^{-07}$	0
	Std	$4.4\;\times \;10^{-112}$	$1.66\;\times \;10^{-87}$	$3.12\;\times \;10^{-16}$	0	$1.58\;\times \;10^{-05}$	0	$3.21\;\times \;10^{-62}$	0	$1.70\;\times \;10^{-51}$	0.085938	0	$2.4\;\times \;10^{-06}$	0

**Table 3:** Contrastive results of the *p*-value Wilcoxon rank-sum test on the benchmark functions.

**Function**	**SCGCRA vs. CPO**	**SCGCRA vs. BKA**	**SCGCRA vs. EHO**	**SCGCRA vs. PO**	**SCGCRA vs. WSA**	SCGCRA vs. HLOA	SCGCRA vs. ECO	SCGCRA vs. IAO	SCGCRA vs. AO	SCGCRA vs. HEOA	SCGCRA vs. NRBO	SCGCRA vs. GCRA
$f_{1}$	1.21$\;\times \;10^{-12}$	1.21$\;\times \;10^{-12}$	1.21$\;\times \;10^{-12}$	N/A	N/A	N/A	1.21$\;\times \;10^{-12}$	N/A	1.21$\;\times \;10^{-12}$	1.21$\;\times \;10^{-12}$	N/A	N/A
$f_{2}$	1.21$\;\times \;10^{-12}$	1.21$\;\times \;10^{-12}$	1.21$\;\times \;10^{-12}$	1.21$\;\times \;10^{-12}$	1.21$\;\times \;10^{-12}$	1.21$\;\times \;10^{-12}$	1.21$\;\times \;10^{-12}$	N/A	1.21$\;\times \;10^{-12}$	1.21$\;\times \;10^{-12}$	4.19$\;\times \;10^{-02}$	N/A
$f_{3}$	1.21$\;\times \;10^{-12}$	1.21$\;\times \;10^{-12}$	1.21$\;\times \;10^{-12}$	N/A	3.33$\;\times \;10^{-02}$	N/A	1.21$\;\times \;10^{-12}$	N/A	1.21$\;\times \;10^{-12}$	1.21$\;\times \;10^{-12}$	N/A	N/A
$f_{4}$	1.21$\;\times \;10^{-12}$	1.21$\;\times \;10^{-12}$	1.21$\;\times \;10^{-12}$	1.21$\;\times \;10^{-12}$	1.21$\;\times \;10^{-12}$	1.21$\;\times \;10^{-12}$	1.21$\;\times \;10^{-12}$	N/A	1.21$\;\times \;10^{-12}$	1.21$\;\times \;10^{-12}$	4.57$\;\times \;10^{-12}$	N/A
$f_{5}$	3.02$\;\times \;10^{-11}$	3.02$\;\times \;10^{-11}$	3.02$\;\times \;10^{-11}$	3.02$\;\times \;10^{-11}$	3.02$\;\times \;10^{-11}$	4.08$\;\times \;10^{-11}$	3.02$\;\times \;10^{-11}$	3.02$\;\times \;10^{-11}$	3.02$\;\times \;10^{-11}$	3.02$\;\times \;10^{-11}$	3.02$\;\times \;10^{-11}$	1.68$\;\times \;10^{-03}$
$f_{6}$	3.02$\;\times \;10^{-11}$	3.02$\;\times \;10^{-11}$	3.02$\;\times \;10^{-11}$	4.85$\;\times \;10^{-03}$	1.21$\;\times \;10^{-12}$	3.02$\;\times \;10^{-11}$	6.52$\;\times \;10^{-09}$	3.02$\;\times \;10^{-11}$	2.37$\;\times \;10^{-10}$	3.02$\;\times \;10^{-11}$	3.02$\;\times \;10^{-11}$	1.10$\;\times \;10^{-08}$
$f_{7}$	1.33$\;\times \;10^{-10}$	3.34$\;\times \;10^{-11}$	3.02$\;\times \;10^{-11}$	3.69$\;\times \;10^{-11}$	8.99$\;\times \;10^{-11}$	3.69$\;\times \;10^{-11}$	3.02$\;\times \;10^{-11}$	6.07$\;\times \;10^{-11}$	3.02$\;\times \;10^{-11}$	3.69$\;\times \;10^{-11}$	3.02$\;\times \;10^{-11}$	4.98$\;\times \;10^{-11}$
$f_{8}$	N/A	N/A	1.20$\;\times \;10^{-12}$	N/A	N/A	N/A	N/A	N/A	N/A	1.21$\;\times \;10^{-12}$	N/A	N/A
$f_{9}$	N/A	N/A	1.21$\;\times \;10^{-12}$	N/A	N/A	N/A	N/A	N/A	2.01$\;\times \;10^{-13}$	N/A	N/A	N/A
$f_{10}$	N/A	N/A	1.92$\;\times \;10^{-09}$	N/A	N/A	N/A	N/A	N/A	8.15$\;\times \;10^{-02}$	1.27$\;\times \;10^{-05}$	N/A	N/A
$f_{11}$	3.02$\;\times \;10^{-11}$	3.02$\;\times \;10^{-11}$	6.10$\;\times \;10^{-01}$	4.64$\;\times \;10^{-02}$	1.11$\;\times \;10^{-06}$	3.02$\;\times \;10^{-11}$	1.07$\;\times \;10^{-07}$	3.02$\;\times \;10^{-11}$	3.83$\;\times \;10^{-05}$	3.02$\;\times \;10^{-11}$	3.02$\;\times \;10^{-11}$	1.00$\;\times \;10^{-03}$
$f_{12}$	3.02$\;\times \;10^{-11}$	3.02$\;\times \;10^{-11}$	N/A	5.39$\;\times \;10^{-01}$	1.11$\;\times \;10^{-06}$	3.02$\;\times \;10^{-11}$	8.89$\;\times \;10^{-10}$	3.02$\;\times \;10^{-11}$	1.20$\;\times \;10^{-08}$	3.02$\;\times \;10^{-11}$	3.02$\;\times \;10^{-11}$	7.60$\;\times \;10^{-07}$
$f_{13}$	3.02$\;\times \;10^{-11}$	1.44$\;\times \;10^{-10}$	7.25$\;\times \;10^{-02}$	1.21$\;\times \;10^{-12}$	4.56$\;\times \;10^{-11}$	9.48$\;\times \;10^{-06}$	4.72$\;\times \;10^{-09}$	1.21$\;\times \;10^{-12}$	1.05$\;\times \;10^{-02}$	1.61$\;\times \;10^{-10}$	3.75$\;\times \;10^{-04}$	1.03$\;\times \;10^{-06}$
$f_{14}$	3.15$\;\times \;10^{-12}$	3.91$\;\times \;10^{-03}$	8.14$\;\times \;10^{-02}$	8.14$\;\times \;10^{-02}$	6.99$\;\times \;10^{-01}$	7.48$\;\times \;10^{-08}$	2.65$\;\times \;10^{-02}$	8.14$\;\times \;10^{-02}$	3.87$\;\times \;10^{-11}$	3.15$\;\times \;10^{-12}$	1.83$\;\times \;10^{-03}$	3.15$\;\times \;10^{-12}$
$f_{15}$	N/A	N/A	1.09$\;\times \;10^{-02}$	3.33$\;\times \;10^{-02}$	N/A	N/A	N/A	N/A	N/A	N/A	N/A	2.21$\;\times \;10^{-06}$
$f_{16}$	2.19$\;\times \;10^{-11}$	1.52$\;\times \;10^{-05}$	2.49$\;\times \;10^{-08}$	6.96$\;\times \;10^{-05}$	1.06$\;\times \;10^{-03}$	2.17$\;\times \;10^{-11}$	1.29$\;\times \;10^{-05}$	1.80$\;\times \;10^{-02}$	1.26$\;\times \;10^{-10}$	2.19$\;\times \;10^{-11}$	5.55$\;\times \;10^{-06}$	2.19$\;\times \;10^{-11}$
$f_{17}$	1.25$\;\times \;10^{-11}$	1.25$\;\times \;10^{-11}$	8.66$\;\times \;10^{-01}$	8.62$\;\times \;10^{-01}$	3.28$\;\times \;10^{-07}$	1.25$\;\times \;10^{-11}$	9.51$\;\times \;10^{-11}$	6.56$\;\times \;10^{-04}$	5.91$\;\times \;10^{-08}$	1.25$\;\times \;10^{-11}$	1.25$\;\times \;10^{-11}$	1.25$\;\times \;10^{-11}$
$f_{18}$	3.02$\;\times \;10^{-11}$	6.39$\;\times \;10^{-12}$	6.66$\;\times \;10^{-03}$	1.41$\;\times \;10^{-11}$	8.38$\;\times \;10^{-08}$	1.32$\;\times \;10^{-04}$	3.79$\;\times \;10^{-10}$	6.59$\;\times \;10^{-01}$	4.87$\;\times \;10^{-05}$	3.02$\;\times \;10^{-11}$	3.04$\;\times \;10^{-06}$	3.03$\;\times \;10^{-03}$
$f_{19}$	3.02$\;\times \;10^{-11}$	4.37$\;\times \;10^{-09}$	8.43$\;\times \;10^{-07}$	1.94$\;\times \;10^{-10}$	5.55$\;\times \;10^{-05}$	1.17$\;\times \;10^{-04}$	8.33$\;\times \;10^{-06}$	6.60$\;\times \;10^{-01}$	6.95$\;\times \;10^{-01}$	3.02$\;\times \;10^{-11}$	5.13$\;\times \;10^{-03}$	2.64$\;\times \;10^{-02}$
$f_{20}$	3.02$\;\times \;10^{-11}$	2.08$\;\times \;10^{-11}$	3.43$\;\times \;10^{-08}$	5.12$\;\times \;10^{-09}$	7.79$\;\times \;10^{-07}$	3.26$\;\times \;10^{-02}$	3.69$\;\times \;10^{-04}$	7.50$\;\times \;10^{-03}$	7.72$\;\times \;10^{-02}$	3.02$\;\times \;10^{-11}$	2.50$\;\times \;10^{-02}$	1.85$\;\times \;10^{-02}$
$f_{21}$	3.02$\;\times \;10^{-11}$	1.21$\;\times \;10^{-12}$	1.21$\;\times \;10^{-12}$	1.21$\;\times \;10^{-12}$	1.21$\;\times \;10^{-12}$	1.21$\;\times \;10^{-12}$	1.21$\;\times \;10^{-12}$	1.21$\;\times \;10^{-12}$	8.34$\;\times \;10^{-03}$	3.50$\;\times \;10^{-09}$	1.21$\;\times \;10^{-12}$	4.03$\;\times \;10^{-02}$
$f_{22}$	N/A	N/A	1.77$\;\times \;10^{-09}$	4.17$\;\times \;10^{-02}$	N/A	N/A	N/A	N/A	N/A	4.95$\;\times \;10^{-06}$	N/A	8.86$\;\times \;10^{-07}$
$f_{23}$	1.21$\;\times \;10^{-12}$	1.21$\;\times \;10^{-12}$	1.20$\;\times \;10^{-12}$	1.21$\;\times \;10^{-12}$	1.21$\;\times \;10^{-12}$	4.57$\;\times \;10^{-12}$	1.21$\;\times \;10^{-12}$	N/A	1.21$\;\times \;10^{-12}$	1.21$\;\times \;10^{-12}$	N/A	1.21$\;\times \;10^{-12}$

**Table 4:** Contrastive results of the three-bar truss design.

Algorithm	Optimum Variables		Optimum Weight
	$A_{1}$	$A_{2}$	
LSHADE [34]	0.785249	0.410335	263.8915
SCA [34]	0.788649	0.408235	263.8715
WOA [34]	0.78860276	0.408453070	263.8958
TEO [34]	0.7886618	0.4082831	263.8958
HGSO [34]	0.778254	0.440528	264.1762
HGS [34]	0.7884562	0.40886831	263.8959
KOA [35]	0.788675	0.408248	263.895843
COA [35]	0.788057	0.410073	263.903379
RUN [35]	0.788793	0.407916	263.895854
SMA [35]	0.788541	0.408627	263.895857
DO [35]	0.788643	0.408339	263.895844
POA [35]	0.788675	0.408248	263.895843
NOA [36]	0.78868	0.40825	263.89584338
GBO [36]	0.78868	0.40825	263.89584338
BKA [2]	0.788675	0.408248	263.895843
SHO [2]	0.788898	0.40762	263.895881
TTAO [18]	0.788688	0.408213	263.8958431
SCHO [37]	0.7886642	0.40827926	263.8958476
APO [38]	0.7887	0.4082	263.89584338
BSLO [39]	0.78867930	0.40823651	263.8958434
FOX [39]	0.78870269	0.4081704	263.8958523
ARSCA [1]	0.7887	0.4081	263.8958
CPO [1]	0.7885	0.4088	263.8959
PKO [40]	0.7886870838	0.4082144942	263.8958435
SFOA [28]	0.78868	0.40825	263.89584
SCGCRA	0.78645	0.41813	263.8543

**Table 5:** Contrastive results of the piston lever design.

Algorithm	Optimum Variables				Optimum Weight
	H	B	X	D	
PSO [41]	133.3	2.44	117.14	4.75	122
DE [41]	129.4	2.43	119.8	4.75	159
GA [41]	250	3.96	60.03	5.91	161
HPSO [41]	135.5	2.48	116.62	4.75	162
CS [42]	0.050	2.043	120	4.085	8.427
SNS [43]	0.050	2.042	120	4.083	8.412698349
SCSO [44]	0.050	2.040	119.99	4.083	8.40901438899551
CSO [44]	0.050	2.399	85.68	4.0804	13.7094866557362
GWO [44]	0.060	2.0390	120	4.083	8.40908765909047
WAO [44]	0.099	2.057	118.4	4.112	9.05943208079399
SSA [44]	0.050	2.073	116.32	4.145	8.80243253777633
GSA [44]	497.49	500	60.041	2.215	168.094363238712
BWO [44]	12.364	12.801	172.02	3.074	95.9980864948937
AOS [45]	0.05	2.042112482	119.951727	4.084004492	8.419142742
GTO [46]	0.05	2.052859	119.6392	4.089713	8.41270
MFO [46]	0.05	2.041514	120	4.083365	8.412698
WOA [46]	0.051874	2.045915	119.9579	4.085849	8.449975
ISA [47]	N/A	N/A	N/A	N/A	8.4
CGO [47]	N/A	N/A	N/A	N/A	8.41281381
MGA [47]	N/A	N/A	N/A	N/A	8.41340665
TTAO [18]	0.05	2.041514	4.083027	120	8.412698323
EGO [29]	1.979653079	3.652740666	426.379188	2.031507236	8.41269886
MVO [29]	0.05	2.046900355	119.92924	4.095582502	8.57509432
ALO [29]	0.05	2.051360067	118.821159	4.102693186	8.53445096
CS-EO [29]	0.05	2.041514	120	4.083027	8.412698
SCGCRA	0.05	0.125364154	120	4.12410157	7.794

**Table 6:** Contrastive results of the gear train design.

**Algorithm**	Optimum Variables				Optimum Cost
	$n_{A}$	$n_{B}$	$n_{C}$	$n_{D}$	
BO [48]	43	19	16	49	2.700857$\;\times \;10^{-12}$
KOA [35]	44	20	16	50	2.700857$\;\times \;10^{-12}$
FLA [35]	44	16	20	49	2.700857$\;\times \;10^{-12}$
COA [35]	23	14	12	48	9.92158$\;\times \;10^{-10}$
RUN [35]	44	17	19	49	2.700857$\;\times \;10^{-12}$
SMA [35]	52	30	13	53	2.307816$\;\times \;10^{-11}$
DO [35]	49	16	19	44	2.700857$\;\times \;10^{-12}$
POA [35]	44	17	19	49	2.700857$\;\times \;10^{-12}$
PDO [47]	48	17	22	54	2.70$\;\times \;10^{-12}$
DMOA [47]	49	19	16	43	2.70$\;\times \;10^{-12}$
AOA [47]	49	19	19	54	2.70$\;\times \;10^{-12}$
CPSOGSA [47]	55	16	16	43	2.31$\;\times \;10^{-11}$
SSA [47]	49	19	19	49	2.70$\;\times \;10^{-12}$
SCA [47]	49	19	34	49	2.700857$\;\times \;10^{-12}$
IEHO [49]	19	16	43	49	2.70085$\;\times \;10^{-12}$
MEWOA [50]	49	16	19	43	2.7099$\;\times \;10^{-12}$
ARO [51]	49	19	16	43	2.7009$\;\times \;10^{-12}$
BCA [52]	43	16	19	49	2.7009$\;\times \;10^{-12}$
BWO [53]	50	18	17	46	7.5421$\;\times \;10^{-17}$
GMO [54]	43	19	16	49	2.700857$\;\times \;10^{-12}$
GBO [18]	53	13	20	34	2.3078$\;\times \;10^{-11}$
TTAO [18]	43	16	19	49	2.70$\;\times \;10^{-12}$
WO [30]	43	16	19	43	2.700857$\;\times \;10^{-12}$
GCRA [6]	55	16	16	43	2.70$\;\times \;10^{-12}$
GOA [6]	49	19	16	43	2.70$\;\times \;10^{-12}$
SCGCRA	50	22	19	52	3.25$\;\times \;10^{-18}$

**Table 7:** Contrastive results of the car side impact design.

**Algorithm**	Optimum Variables						Optimum Weight
	$x_{1}$	$x_{2}$	$x_{3}$	$x_{4}$	$x_{5}$	$x_{6}$	
	$x_{7}$	$x_{8}$	$x_{9}$	$x_{10}$	$x_{11}$		
ACO [55]	0.5	1.12004	0.5	1.29627	0.5	1.5	
	0.5	0.345	0.192	−18.905	−0.0008		22.84371
KH [55]	0.5	1.14747	0.5	1.26118	0.5	1.5	
	0.5	0.345	0.345	−13.998	−0.8984		22.88596
HHO [55]	0.5	1.15627	0.5	1.27133	0.5	1.4777	
	0.5	0.345	0.192	−14.592	−2.4898		22.98537
BOA [55]	0.8246	1.03224	0.54007	1.35639	0.6377	1.26889	
	0.5854	0.192	0.345	−5.7333	0.4352		25.06573
HGSO [55]	0.5	1.22375	0.5	1.27111	0.5	1.31085	
	0.5	0.345	0.345	−4.3235	2.93676		23.43457
LIACO [55]	0.5	1.11593	0.5	1.30293	0.5	1.5	
	0.5	0.192	0.345	−19.64	−0.000003		22.84299
SMO [55]	0.5	1.11634	0.5	1.30224	0.5	1.5	
	0.5	0.345	0.345	−19.566	0.000001		22.84298
DOA [56]	0.5081	1.2021	0.5318	1.3052	0.5719	1.4954	
	0.5557	0.303	0.2585	−24.8171	3.4047		23.9682
DCS [56]	0.5772	1.2586	0.5195	1.2002	0.5463	1.258	
	0.5073	0.278	0.2669	2.0888	5.4035		23.9995
COA [56]	0.5	1.2791	0.5	1.2739	1.2828	0.5	
	0.5	0.2954	0.192	3.557	19.0792		25.2083
MSA [56]	0.5151	1.2684	0.5545	1.3737	0.5261	1.3484	
	0.7156	0.2869	0.2167	−7.2394	11.7869		25.2334
HLOA [56]	0.5	1.0669	0.8016	1.0704	0.504	1.4873	
	0.5	0.192	0.192	−29.9786	3.2119		23.6956
AROA [56]	0.5	1.5	0.5	1.2928	0.5	0.5	
	0.5	0.192	0.3195	8.8265	23.0874		25.3642
EGO [29]	0.5	1.1107	0.5	1.312	0.5001	1.5	
	0.50001	0.98732	0.04604	−20.57	0.18084		22.84570
MVO [29]	0.5	1.1352	0.5012	1.27318	0.5003	1.5	
	0.50403	0.53489	0.23089	−16.1449	0.99051		22.88565
ETO [57]	0.50282	1.2414	0.51604	1.2201	0.60334	1.3878	
	0.5	0.74832	0.06747	2.2526	−7.2818		23.2574
SCHO [57]	0.5	1.10286	0.87088	0.88643	0.52609	1.49992	
	0.5	0.03508	0.19439	−30	−0.5913		23.7209
AOA [57]	0.5	1.2279	0.5	1.4332	0.5	1.5	
	0.5	0.61018	0.21619	0.00126	−0.0765		24.1125
HGS [57]	0.5	1.10612	1.11044	0.5	0.5	1.5	
	0.5	4.4$\;\times \;10^{-09}$	0.00000	−30	−6.0 $\times \;10^{-09}$		23.8188
GJO [57]	0.5	1.20309	0.50327	1.28778	0.51053	1.5	
	0.5	0.00000	9.5$\;\times \;10^{-05}$	−22.115	−0.0536		23.4052
ROA [31]	1.098334 901	0.957459058	1.112521155	1.043356648	0.730817433	1.009550656	
	0.51561597	0.345	0.345	0.053235933	0.042350889		28.40584747
SCSO [31]	0.502366774	1.23533939	0.5	1.223008761	0.515267967	1.39187245	
	0.50003369	0.340647775	0.211950171	1.374158706	−7.77399175		23.35787723
SHO [31]	1.5	1.267885192	1.5	0.768783364	1.11811662	0.74785158	
	0.56089667	0.345	0.345	2.050521688	3.263049114		34.86111849
SOA [31]	0.500139239	1.254868587	0.5	1.205871077	0.739233716	0.772309974	
	0.5	0.316999014	0.30308334	0.749660043	2.039711514		23.8070425
SFOA [28]	0.5	1.234	0.5	1.187	0.875	0.892	
	0.4	0.345	0.192	1.5	0.572		23.5616
SCGCRA	0.5	1.11643	0.5	1.30208	0.5	1.5	
	0.5	0.345	0.192	−19.54935	−0.00431		22.84294

**Table 8:** Contrastive results of the multiple-disk clutch brake design.

**Algorithm**	Optimum Variables					Optimum Weight
	$r_{i}$	$r_{0}$	t	F	Z	
TLBO [58]	70	90	1	810	3	0.313657
MFO [59]	70	90	1	910	3	0.313656
NSGA-II [60]	70	90	1.5	1000	3	0.470400
MVO [61]	70	90	1	910	3	0.313656
CMVO [61]	70	90	1	910	3	0.313656
WCA [62]	70	90	1	910	3	0.313656
APSO [63]	76	96	1	840	3	0.337181
IAPSO [63]	70	90	1	900	3	0.31365661
DAPSO-GA [63]	70	90	1	1000	3	0.31365661
FSO [64]	70	90	1	870	3	0.31365661053
GOA [65]	71	92	1	835	3	0.3355146
EOBL-GOA [65]	70	90	1	984	3	0.31365661053
ABC [66]	70	90	1	790	3	0.313657
PSO [66]	70	90	1	860	3	0.3136566
CS [66]	70	90	1	810	3	0.3136566
GSA [66]	72	92	2	815	3	0.3175771
AEO [66]	70	90	1	810	3	0.3136566
AHA [67]	70	90	1	840	3	0.3136566
HBO [68]	70	90	1	1000	3	0.3136566
HGS [69]	70	90	1	1000	3	0.313657
I-ABC [70]	70	90	1	900	3	0.313766
MRFO [71]	70	90	1	835	3	0.3136566
GA [71]	72	92	1	918	3	0.321498
DE [71]	71	92	1	835	3	0.3355146
RSO [32]	70	90	1	810	3	0.313657
SCGCRA	70	90	1	600	2	0.235247

**Table 9:** Contrastive results of the rolling element bearing design.

**Algorithm**	Optimum Variables					Optimum Cost
	$D_{m}$	$D_{b}$	Z	$f_{i}$	$f_{o}$	
	$K_{D\min}$	$K_{D\max}$	ε	e	ζ	
HHO [33]	125	21	11.09207	0.515	0.515	
	0.4	0.6	0.3	0.050474	0.6	83,011.88
RSA [33]	125.1722	21.29734	10.88521	0.515253	0.517764	
	0.41245	0.632338	0.301911	0.024395	0.6024	83,486.64
RSO [72]	125	21.41769	10.94027	0.515	0.515	
	0.4	0.7	0.3	0.02	0.6	85,069.021
EPO [63]	125	21.4189	10.94113	0.515	0.515	
	0.4	0.7	0.3	0.02	0.6	85,067.983
ESA [63]	125	21.4175	10.94109	0.51	0.515	
	0.4	0.7	0.3	0.02	0.6	85,070.085
HSCAHS [63]	125	10.5	4	0.515	0.515	
	0.4	0.6	0.3	0.02	0.6	85,539.192
SSA [63]	125	20.77562	11.01247	0.515	0.515	
	0.5	0.61397	0.3	0.05004	0.61001	82,773.982
PSOGSA [73]	125.008533	21.112638	11.062267	0.515	0.5195993	
	0.40487643	0.6032501	0.3	0.1	0.7037127	83,650.9164
WOA [73]	125.100734	21.4233	10.95119	0.515	0.515	
	0.4	0.7	0.314216	0.02	0.6	85,265.167
HGSA [73]	125.708006	21.4233005	10.999978	0.515	0.515	
	0.5	0.7	0.300304	0.0271098	0.6	85,532.7227
ACVO [73]	125.70959	21.4232997	11.000104	0.515	0.515	
	0.48352698	0.61821897	0.3002753	0.02	0.6478817	85,533.4103
AFT [74]	125	21.418	11.356	0.515	0.515	
	0.4	0.68	0.3	0.02	0.622	85,206.641
AHA [67]	125.718411	21.42535	10.527979	0.515	0.515155	
	0.470216	0.640818	0.300012	0.095122	0.682241	85,547.49822
HBO [68]	125.7227184	21.4233	11	0.515	0.515	
	0.438476	0.699998	0.3	0.047532	0.601081	85,533.18
HPO [70]	125	21.875	10.777	0.515	0.515	
	0.4	0.7	0.3	0.029	0.6	83,918.4925
MRFO [71]	125.7190556	21.4255902	11	0.515	0.515	
	0.4050856	0.6905778	0.3	0.0536602	0.6925802	85,549.239
CS [71]	125.442787	21.205159	11	0.515	0.5416852	
	0.5	0.7	0.3	0.0975781	0.6015492	83,988.259
RUN [75]	125.2142	21.59796	11.4024	0.515	0.515	
	0.40059	0.61467	0.3053	0.02	0.63665	83,680.47
ARO [51]	125.7189	21	10.5403	0.515	0.515	
	0.4459	0.672132	0.3	0.0825	0.6317	85,548.5106
MGA [76]	125.718	21.8745119	10.7770658	0.51500082	0.51500299	
	0.405908353	0.65558802	0.30000415	0.07754492	0.6	83,912.87983
CGO [76]	125	21.875	10.777009	0.515	0.515	
	0.4	0.64620052	0.3	0.050152445	0.6	83,918.49253
EVO [76]	125.7190556	21.4255902	10.6955328	0.515	0.515	
	0.463182936	0.6999265	0.3	0.063431519	0.604213108	81,859.7415974
SELO [77]	126.3521	21.0299	11	0.515	0.515	
	0.4	0.6011	0.3	0.1	0.6004	83,805.29
LFD [77]	126.3999	21	11	0.515	0.5251	
	0.5	0.6	0.3	0.1	0.6	83,670.78
SETO [77]	125.7227	21.4233	11	0.515	0.515	
	0.4	0.7	0.3	0.1	0.6	85,539.19
SCGCRA	126.2339	20.1947	10.5139	0.5524	0.5428	
	0.4072	0.6565	0.3254	0.0681	0.6142	90,020.39

## Data Availability

The data presented in this study are available upon request from the corresponding author. The MATLAB code developed for this study is available from the corresponding author upon reasonable request.
